# Circulating metabolic profile in idiopathic pulmonary fibrosis: data from the IPF-PRO Registry

**DOI:** 10.1186/s12931-023-02644-7

**Published:** 2024-01-25

**Authors:** Ross Summer, Jamie L. Todd, Megan L. Neely, L. Jason Lobo, Andrew Namen, L. Kristin Newby, Shirin Shafazand, Sally Suliman, Christian Hesslinger, Sascha Keller, Thomas B. Leonard, Scott M. Palmer, Olga Ilkayeva, Michael J. Muehlbauer, Christopher B. Newgard, Jesse Roman

**Affiliations:** 1 Thomas Jefferson University, Philadelphia, PA USA; 2 Duke Clinical Research Institute, Durham, NC USA; 3 Duke University Medical Center, Durham, NC USA; 4 University of North Carolina School of Medicine, Chapel Hill, NC USA; 5 Wake Forest School of Medicine, Winston-Salem, NC USA; 6 University of Miami, Miami, FL USA; 7 Banner University Medical Center, Phoenix, AZ USA; 8 Boehringer Ingelheim Pharma GmbH & Co. KG, Biberach, Germany; 9 Boehringer Ingelheim Pharmaceuticals, Inc, Ridgefield, CT USA; 10 Duke Molecular Physiology Institute, Durham, NC USA; 11 Department of Medicine, Division of Endocrinology, Metabolism, and Nutrition, Duke University School of Medicine, Durham, NC USA; 12 Jane and Leonard Korman Institute, Thomas Jefferson University, Philadelphia, PA USA

**Keywords:** Biomarkers, Interstitial lung diseases, Metabolomics, Pulmonary fibrosis

## Abstract

**Background:**

The circulating metabolome, reflecting underlying cellular processes and disease biology, has not been fully characterized in patients with idiopathic pulmonary fibrosis (IPF). We evaluated whether circulating levels of metabolites correlate with the presence of IPF, with the severity of IPF, or with the risk of clinically relevant outcomes among patients with IPF.

**Methods:**

We analyzed enrollment plasma samples from 300 patients with IPF in the IPF-PRO Registry and 100 individuals without known lung disease using a set of targeted metabolomics and clinical analyte modules. Linear regression was used to compare metabolite and clinical analyte levels between patients with IPF and controls and to determine associations between metabolite levels and measures of disease severity in patients with IPF. Unadjusted and adjusted univariable Cox regression models were used to evaluate associations between circulating metabolites and the risk of mortality or disease progression among patients with IPF.

**Results:**

Levels of 64 metabolites and 5 clinical analytes were significantly different between patients with IPF and controls. Among analytes with greatest differences were non-esterified fatty acids, multiple long-chain acylcarnitines, and select ceramides, levels of which were higher among patients with IPF versus controls. Levels of the branched-chain amino acids valine and leucine/isoleucine were inversely correlated with measures of disease severity. After adjusting for clinical factors known to influence outcomes, higher levels of the acylcarnitine C:16-OH/C:14-DC were associated with all-cause mortality, lower levels of the acylcarnitine C16:1-OH/C14:1DC were associated with all-cause mortality, respiratory death, and respiratory death or lung transplant, and higher levels of the sphingomyelin d43:2 were associated with the risk of respiratory death or lung transplantation.

**Conclusions:**

IPF has a distinct circulating metabolic profile characterized by increased levels of non-esterified fatty acids, long-chain acylcarnitines, and ceramides, which may suggest a more catabolic environment that enhances lipid mobilization and metabolism. We identified select metabolites that were highly correlated with measures of disease severity or the risk of disease progression and that may be developed further as biomarkers.

**Trial registration:**

ClinicalTrials.gov; No: NCT01915511; URL: www.clinicaltrials.gov.

**Supplementary Information:**

The online version contains supplementary material available at 10.1186/s12931-023-02644-7.

## Background

Idiopathic pulmonary fibrosis (IPF) is a progressive and ultimately fatal interstitial lung disease [1]. IPF is characterized by progressive decline in lung function due to the excess deposition of extracellular matrix components, which ultimately leads to destruction of the lung architecture and respiratory failure [2]. Epithelial cell injury with activation of fibroblasts and production of pro-fibrotic growth factors is considered pathogenic [3]. Two anti-fibrotic drugs, nintedanib and pirfenidone, slow decline in lung function in patients with IPF [4, 5], and have been licensed for its treatment, but the disease remains progressive. The rate of disease progression among patients with IPF is variable [6, 7] and while some circulating biomarkers have shown promise as predictors of progression [8–11], for an individual patient, it remains challenging to predict the rate at which their disease will progress.

As metabolites represent the end products of cellular processes and are potential effectors of disease biology, the circulating metabolome may provide novel insights into disease activity and identify candidate biomarkers of disease progression. This idea is supported by studies suggesting that the pathogenesis of pulmonary fibrosis is associated with metabolic abnormalities including mitochondrial dysfunction; lipid dysregulation; elevations in lactic acid and lactate dehydrogenase 5; alterations in glycolysis, glutathione biosynthesis, adenosine triphosphate degradation, and ornithine aminotransferase pathways [12–20]. The circulating metabolome in patients with IPF has not been extensively studied, but associations have been identified between select metabolites and measures of disease severity and the risk of progression [20, 21]. In this study, we quantified the circulating metabolome in patients with IPF and controls without known lung disease to identify metabolites present at different levels between the groups. We then related circulating metabolite levels to measures of disease severity in the patients with IPF and evaluated their utility for predicting the risk of clinically relevant outcomes among these patients.

## Methods

### Study population

The IPF cohort was drawn from the Idiopathic Pulmonary Fibrosis PRospective Outcomes (IPF-PRO) Registry, a multicenter observational registry of patients with IPF that was diagnosed or confirmed at the enrolling center in the past 6 months [22]. The cohort comprised 300 patients, enrolled between June 2014 and February 2017, who had a blood sample and data on critical clinical variables at enrollment, including diagnostic criteria for IPF (definite, probable, possible) according to the 2011 international guidelines [23].

The control cohort was drawn from the Measurement to Understand the Reclassification of Disease of Cabarrus/Kannapolis (MURDOCK) Study, a registry of adult residents in North Carolina in which self-reported health information and biological samples are collected [24]. To ensure the control cohort had a similar age, race, and ethnicity distribution to the IPF cohort, controls were White, non-Hispanic and aged 60 to 80 years. Participants were excluded if they had self-reported respiratory disease, cancer, or autoimmune disease, were active smokers, had active second-hand tobacco exposure, or used respiratory-targeted medications or immunomodulators. Random sampling with stratification by sex and smoking status (ever versus never) was used to select 100 controls with a similar distribution of these characteristics to the IPF cohort.

The IPF-PRO Registry was approved by the Duke University Institutional Review Board (Pro00046131). The IPF-PRO Registry protocol was also approved by the relevant Institutional Review Boards and/or local Independent Ethics Committees prior to enrollment at each site (listed in the Acknowledgments). All patients provided written informed consent. The IPF-PRO Registry was registered with ClinicalTrials.gov (Identifier: NCT01915511). The MURDOCK Study Community Registry and Biorepository was approved by the Duke University Health Institutional Review Board (Pro00011196) and all participants provided written informed consent. The MURDOCK Study Community Registry and Biorepository was registered with ClinicalTrials.gov (Identifier: NCT01708408).

### Metabolite quantification

A set of metabolites comprising 15 amino acids, 45 acylcarnitines, 21 ceramides, 34 sphingomyelins, 3 branched chain keto-acids, and 3-hydroxyisobutyrate was quantified in plasma samples taken at enrollment using flow-injection tandem mass spectrometry (MS/MS) [25, 26] or liquid chromatography(LC)-MS/MS as previously described [27–30]. Quantitative measurements were achieved by adding known quantities of stable isotope-labeled internal standards to the biological samples. The assays were run in a 96-well-plate format, with a calibration curve and a set of two quality-control samples at the beginning and end of each plate. An ion ratio of the analyte respective to the internal standard was computed from centroided spectra, and ion ratios converted to concentrations using the calibration curve slope; this allowed values to be analyzed even if below the limit of quantification. Values of 0 were imputed as half the lowest detectable value for a given analyte.

To provide context for interpretation of the metabolite data, several common clinical analytes were measured, using published methods [28, 29], in each sample: total, high-density lipoprotein (HDL) and low-density lipoprotein (LDL) cholesterol, glucose, ketones, lactate, non-esterified fatty acids (NEFA), triglycerides, glycerol, and 3-hydroxybutyrate. There was insufficient sample volume to permit measurement of 3-hydroxybutyrate in 9.2%, HDL cholesterol in 4.0%, LDL cholesterol in 1.0% and ketones in 0.2% of samples. For analyses of clinical analytes, only cases and controls with complete data for all analytes were included. Metabolite and clinical analyte concentrations were log_2_ transformed prior to analysis.

### Statistical analyses

Descriptive statistics were used to analyze patient characteristics and the level of each metabolite or clinical analyte in patients with IPF and controls. Linear regression was used to assess whether metabolite or clinical analyte concentrations differed by IPF or control status. *P* values were corrected for multiple comparisons using the Benjamini-Hochberg procedure to control the false discovery rate (FDR) at 5%. Comparisons were considered statistically significant if the FDR-corrected *P* < 0.05 and clinically significant if the absolute fold-change ≥ 30% (i.e. absolute log_2_ fold-change ≥ 0.38).

Among patients with IPF, linear regression models were used to determine the associations between circulating metabolites and three clinical measures of disease severity at enrollment: forced vital capacity (FVC) % predicted, diffusion capacity of the lung for carbon monoxide (DLco) % predicted, and composite physiologic index (CPI) [31]. Each measure was analyzed as a continuous variable. Comparisons were considered statistically significant if the FDR-corrected *P* < 0.05 and clinically significant if  ≥ 5-unit difference in the disease severity measure per unit change in the log_2_ uM concentration of the metabolite (i.e. if a doubling of the metabolite concentration was associated with a ≥ 5-point difference in the disease severity measure).

To identify candidate biomarkers of clinically relevant outcomes among patients with IPF, univariable associations between levels of metabolites or clinical analytes at enrollment and outcomes were determined using Cox proportional hazards regression analyses. We present results for all-cause death; respiratory death; composite of all-cause death or lung transplantation; composite of respiratory death or lung transplantation; composite of decline in FVC ≥ 10% predicted, death, or lung transplantation; composite of decline in DLco ≥ 15% predicted, death, or lung transplantation. Kaplan-Meier plots were used to describe cumulative event probabilities. Analyses were unadjusted and adjusted for clinical factors known to influence outcomes i.e., sex, age, FVC % predicted, DLco % predicted, supplemental oxygen use (all assessed at enrollment). Comparisons were considered statistically significant if the FDR-corrected *P* < 0.05 and clinically significant if the hazard ratio was < 0.67 or > 1.5. The linearity and proportional hazards assumptions were assessed. For metabolites for which the linearity assumption failed, piece-wise linear splines with 1 or 2 knots were used to characterize the non-linearity and hazard ratios with 95% confidence intervals for each segment are presented to describe the relationship between the metabolite and outcome. For metabolites for which the proportional hazards assumption failed, an interaction term with time was included in the model as a time-dependent covariate and hazard ratios with 95% confidence intervals at 6, 12, and 24 months are presented to describe how the association changed during follow-up.

## Results

### Cohort characteristics

The characteristics of the IPF and control cohorts at enrollment are shown in Table 1. In the IPF cohort, the median (Q1, Q3) age was 70.0 (65.0, 75.0) years, 74.7% were men, 93.7% were White and 67.3% were former smokers (Table 1). Most patients (74.0%) were classified by the investigator as having definite IPF. Most (56.0%) were taking nintedanib or pirfenidone. Median (Q1, Q3) FVC % predicted was 69.7 (61.0, 80.2), DLco % predicted was 40.5 (31.6, 49.4) and CPI was 53.7 (46.6, 60.6). In the control cohort, the median (Q1, Q3) age was 66.0 (63.0, 71.5) years, 74.0% were men, all were White, and 68.0% were former smokers. The proportion of IPF and control cohorts reporting use of statins was similar (53.3% and 50.0%, respectively), but more patients in the IPF cohort were taking H2 blockers, anticoagulants, bronchodilators, and insulin.

**Table 1:** Clinical characteristics of the IPF and control cohorts at enrollment

	IPF cohort (n = 300)	Control cohort (n = 100)
Age, years	70.0 (65.0, 75.0)	66.0 (63.0, 71.5)
Male	224 (74.7)	74 (74.0)
Hispanic/Latino Ethnicity	8 (2.7)	0
Race		
White	281 (93.7)	100 (100.0)
Black/African-American	8 (2.7)	0
Asian	6 (2.0)	0
Other	5 (1.7)	0
Smoking status		
Past	202 (67.3)	68 (68.0)
Never	96 (32.0)	32 (32.0)
Current	2 (0.7)	0
BMI, kg/m^2^	29.3 (26.3, 32.8)	27.7 (24.6, 31.9)
Diagnostic criteria*		
Definite IPF	222 (74.0)	–
Possible IPF	16 (5.3)	–
Probable IPF	62 (20.7)	–
Emphysema on HRCT	31 (10.3)	–
FEV_1_% predicted	77.3 (67.9, 89.1)	–
FVC % predicted	69.7 (61.0, 80.2)	–
FEV_1_/FVC ratio	74.0 (72.7, 75.5)	–
DL_CO_ % predicted	40.5 (31.6, 49.4)	–
CPI	53.7 (46.6, 60.6)	–
Medical history		
Coronary artery disease	92 (30.7)	22 (22.0)
Atrial fibrillation	37 (12.3)	13 (13.0)
Diabetes	58 (19.3)	28 (28.0)
Medication use^†^		
Statins^‡^	153 (53.3)	50 (50.0)
Proton pump inhibitors^§^	173 (60.5)	21 (21.0)
Angiotensin converting enzyme inhibitors / angiotensin receptor blockers^||^	68 (23.9)	48 (48.0)
Pirfenidone	111 (37.0)	–
Bronchodilators^§^	87 (30.4)	–
Anti-coagulants^§^	59 (20.6)	14 (14.0)
Nintedanib	57 (19.0)	–
Oral steroids^§^	39 (13.6)	–
H2 blockers^||^	36 (12.6)	3 (3.0)
Insulin^§^	24 (8.4)	4 (4.0)
Pulmonary vasodilators^||^	8 (2.8)	–
N-acetylcysteine^¶^	9 (3.2)	–
Immunosuppressants/cytotoxic drugs^||^	3 (1.1)	–
Supplemental oxygen at rest^#^	61 (20.4)	–

*Definition of abbreviations: BMI* body mass index, *FEV*_*1*_ forced expiratory volume in 1 s, *FVC* forced vital capacity, *DLco* diffusion capacity of the lung for carbon monoxide, *CPI* composite physiologic index. Data are presented as No. (%) or median (25th, 75th percentile). *Based on 2011 American Thoracic Society, European Respiratory Society, Japanese Respiratory Society, and Latin American Thoracic Society diagnostic guidelines. ^†^Medication use in the IPF cohort was determined via chart abstraction; in the control cohort, medication use was self-reported and then categorized to facilitate comparative description. ^‡^No. (%) of 287 patients with available data. ^§^No. (%) of 286 patients with available data. ^||^No. (%) of 285 patients with available data. ^¶^No. (%) of 284 patients with available data. ^#^No. (%) of 299 patients with available data

### Associations between metabolites and IPF

**Fig. 1:**
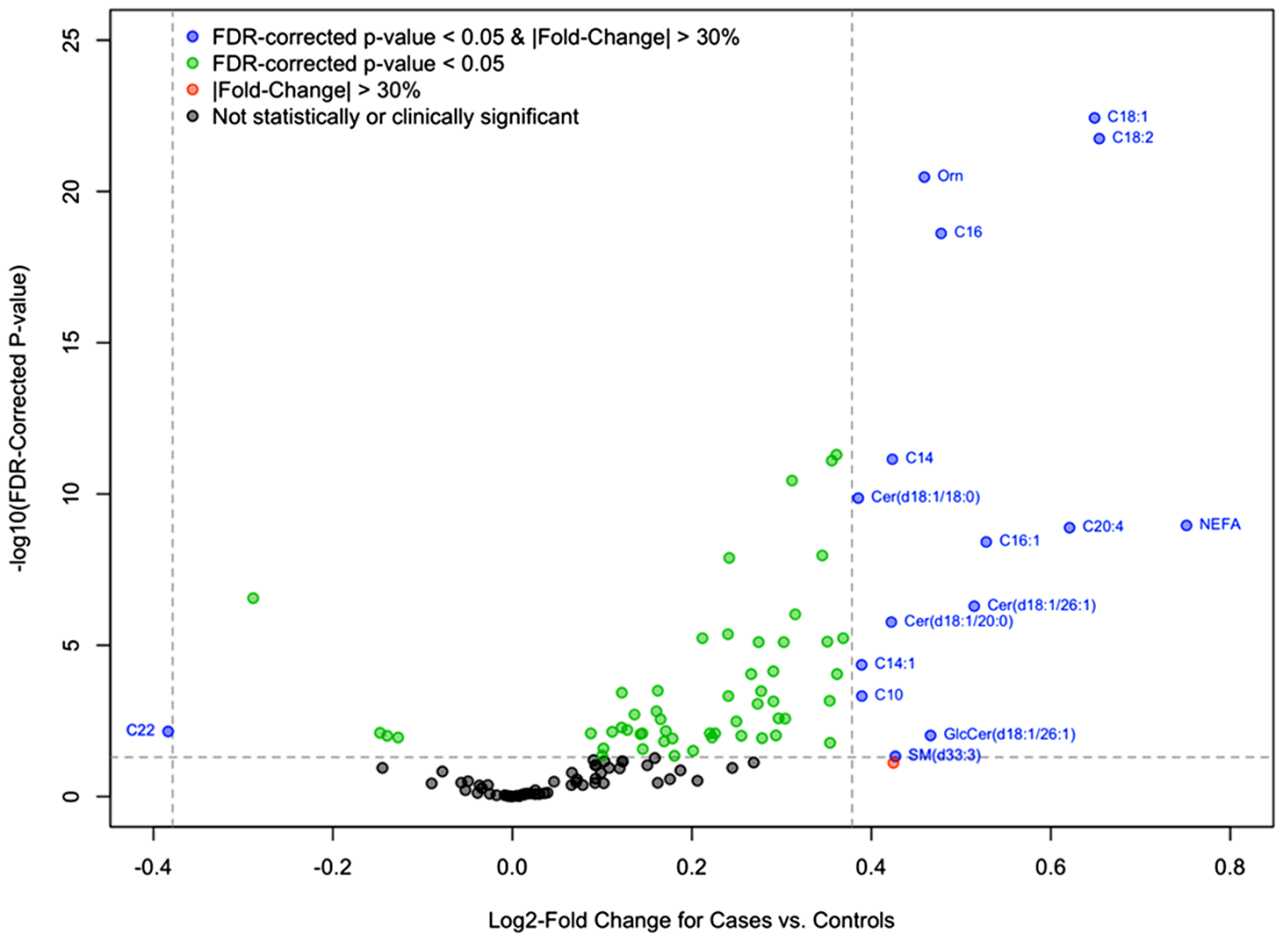
Associations between circulating metabolites and clinical analytes in patients with IPF vs. controls

The concentrations of metabolites and clinical analytes in the IPF and control cohorts are given in Tables S1 and S2 in Additional file 1. Linear regression analyses identified 64 metabolites and 5 clinical analytes with significantly different levels between patients with IPF and controls. Sixteen of these had an absolute fold-change ≥ 30% (log_2_ fold-change ≥ 0.38) between groups and four had an absolute fold-change ≥ 50% (log_2_ fold-change ≥ 0.58) between groups (Fig. 1; Tables S3 and S4 in Additional file 1). Levels of these metabolites/clinical analytes were not significantly different between patients with IPF who were versus were not using anti-fibrotic therapy at enrollment (Table S5 in Additional file 1). Among the clinical analytes and metabolites with the greatest difference between groups were NEFA, the amino acid ornithine, multiple long chain acylcarnitines and select ceramides, with higher levels seen in the patients with IPF relative to the controls (Fig. 1).

### Circulating metabolites and IPF severity

Associations between circulating metabolites or clinical analytes and disease severity measures among patients with IPF are shown in Tables S6–S8 in Additional file 1. Metabolites or clinical analytes with statistically and/or clinically significant associations with disease severity measures are summarized in Table 2. Higher levels of several amino acids, including the branched-chain amino acids valine and leucine/isoleucine, were associated with less severe disease based on DLco % predicted, FVC % predicted, and/or CPI (Table 2). Higher levels of select acylcarnitines (C8, C10:1, C10, C10-OH/C8-DC, C14:2), ceramides (d18:1/16:0, glucosylceramide[d18:1/16:0]) and sphingomyelins (d34:1, d42:3, d32:1) were generally associated with more severe disease (Table 2). No notable differences were observed in analyses adjusted for anti-fibrotic drug use at enrollment (Tables S6–S8 in Additional file 1).

**Table 2:** Association of circulating metabolites or clinical analytes with disease severity measures at enrollment in patients with IPF in unadjusted analyses. Analytes meeting statistical significance (FDR-corrected *P* < 0.05) or clinical significance (≥ 5-unit difference in disease severity measure per unit change in log_2_ uM of the metabolite concentration) thresholds are shown

Analyte	Effect estimate*	FDR-corrected *P* value
***DLco % predicted***		
Valine	8.65	0.049
Leucine/isoleucine	6.93	0.049
Histidine	8.12	0.093
Tyrosine	5.84	0.093
Glx	5.34	0.093
C8	-2.88	0.049
C10:1	-4.24	0.033
C10	-2.67	0.049
C10-OH/C8-DC	-3.96	0.049
C14:2	-3.09	0.049
Cer(d18:1/16:0)	-6.50	0.049
GlcCer(d18:1/16:0)	-4.95	0.049
SM(d34:1)	-8.62	0.050
SM(d42:3)	-5.32	0.093
***FVC % predicted***		
Ornithine	5.57	0.398
SM(d32:1)	5.09	0.430
***CPI***		
Valine	-6.80	0.067
Leucine/isoleucine	-5.56	0.089
Histidine	-6.48	0.096
SM(d34:1)	5.19	0.171

*Definition of abbreviations: FDR* false discovery rate, *DLco* diffusion capacity of the lung for carbon monoxide, *FVC* forced vital capacity, *CPI* composite physiologic index. *Observed unit difference in the disease severity measure per unit change in log_2_ uM of metabolite concentration (e.g. doubling of metabolite concentration)

### Circulating metabolites and outcomes

**Fig. 2:**
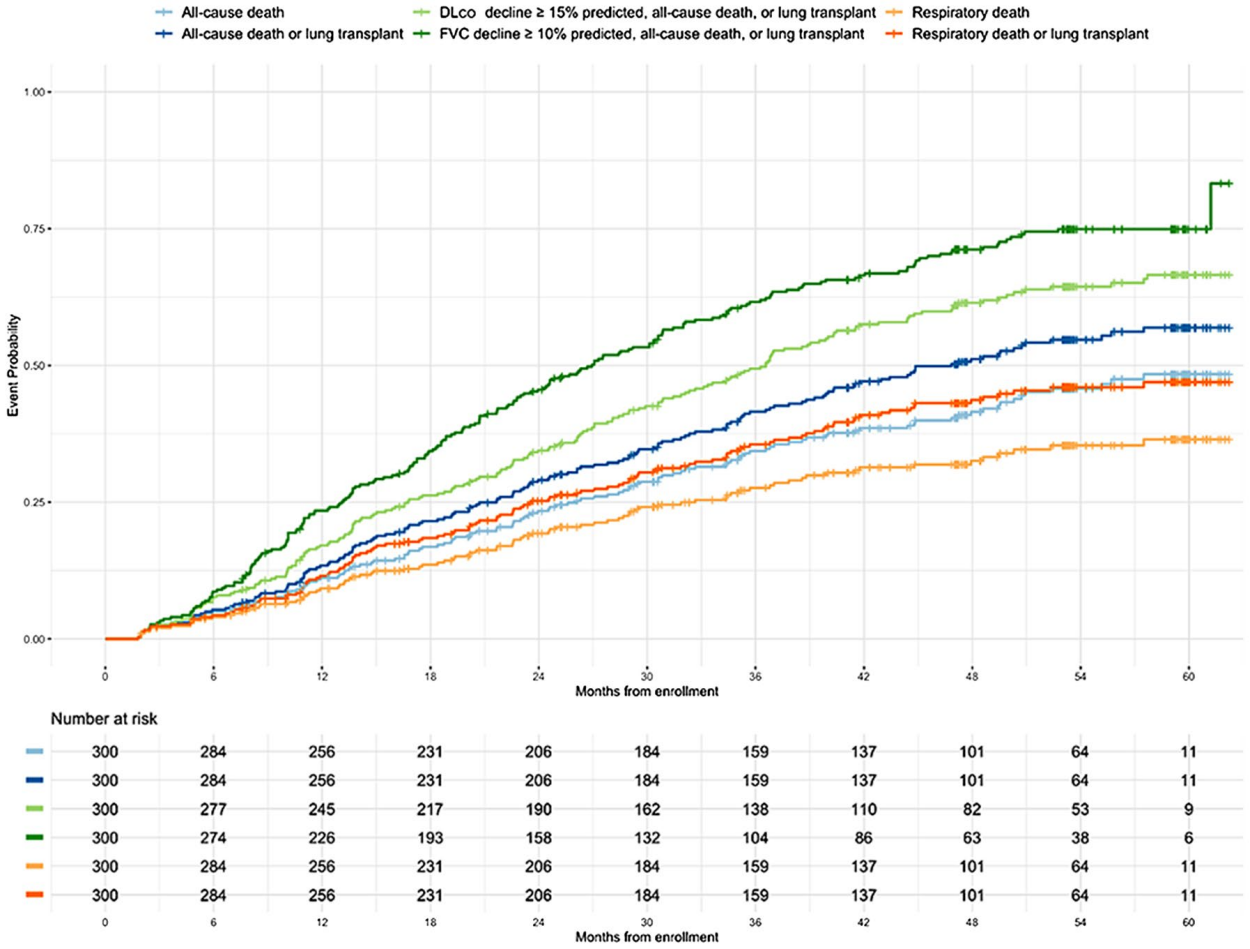
Cumulative probability of an event of outcomes over follow-up

**Fig. 3:**
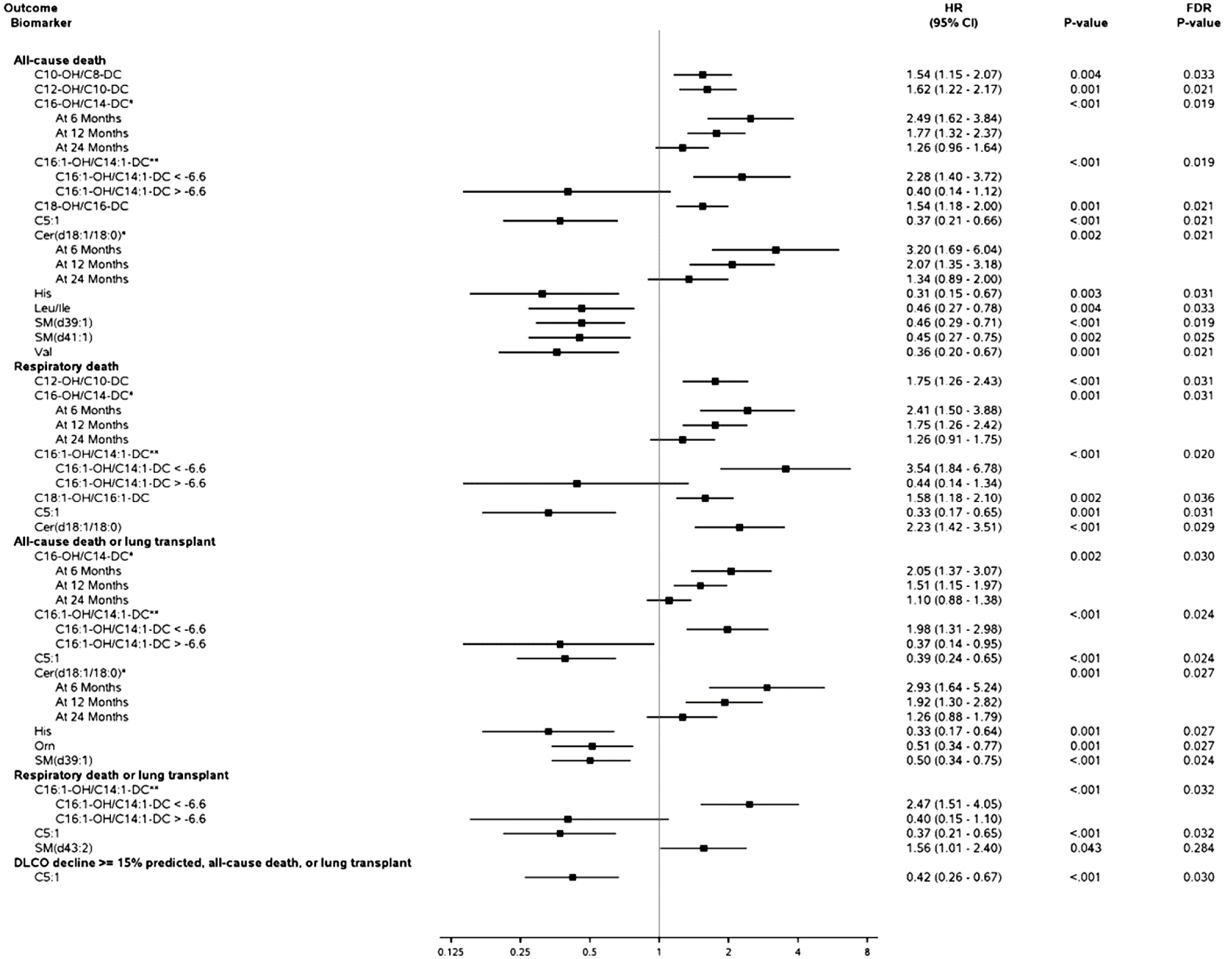
Unadjusted univariate association between circulating metabolites and risk of outcomes. Metabolites meeting statistical significance (FDR-corrected *P* < 0.05) or clinical significance (hazard ratio < 0.67 or > 1.5) thresholds in unadjusted or adjusted analyses are shown. *Metabolite failed proportional hazards assumptions so hazard ratios at 6, 12, and 24 months are shown. **There was a non-linear relationship between metabolite and outcome so a piecewise linear spline was used; a hazard ratio is shown for each segment

Outcomes were assessed over a median (25th, 75th percentile) follow-up period of 39.9 (20.1, 53.3) months. The cumulative event probability of each outcome is shown in Fig. 2. In unadjusted analyses, select amino acids, acylcarnitines, ceramides and sphingomyelins were significantly associated with outcomes (Fig. 3). After adjusting for clinical factors, higher levels of the acylcarnitine C:16-OH/C:14-DC remained significantly associated with all-cause mortality, lower levels of the acylcarnitine C16:1-OH/C14:1DC remained significantly associated with all-cause mortality, respiratory death, and respiratory death or lung transplant, and higher levels of the sphingomyelin d43:2 were associated with the risk of respiratory death or lung transplantation (Fig. E1 in Additional file 1).

## Discussion

We found that the peripheral blood metabolome of patients with IPF was distinct from that of individuals without known lung disease of similar age and sex distribution. In addition, select metabolites were highly correlated with measures of disease severity and with the risk of clinically relevant outcomes, even after accounting for clinical factors known to impact such outcomes.

In our study, the patients with IPF had significantly higher circulating levels of several lipid species, including multiple long-chain acylcarnitines and ceramides and one sphingomyelin species, compared with the controls. These findings are consistent with data showing dysregulated fatty acid or lipid metabolism in mouse models of IPF [16, 32], elevated free fatty acids or dysregulation of fatty acid metabolism-related genes in the lungs of patients with IPF [16, 33], and elevated circulating levels of fatty acids in patients with IPF [19, 21, 34]. The pattern of higher levels of NEFA along with higher levels of acylcarnitines and select ceramides in patients with IPF in our study points to enhanced availability of fatty acids, possibly due to lipolysis related to wasting. An increase in lipolysis is supported by the observation that mobilization of fatty acids from adipose triacylglycerol stores is accompanied by release of the glycerol backbone upon which the fatty acids were esterified, and glycerol levels were higher in the plasma of patients with IPF compared with controls. Higher NEFA levels may also be a marker of impaired action of insulin to suppress lipolysis (insulin resistance) [35], although this seems less likely because the patients with IPF had lower circulating glucose levels and a smaller proportion had diabetes than the controls. As anti-fibrotic treatments are associated with gastrointestinal side effects [4, 5], we considered whether the derangements in circulating lipids might be related to weight loss and/or malabsorption, but among the lipid metabolites with higher levels in patients with IPF, we did not find differences in levels between patients with IPF who were and were not using anti-fibrotic therapy. Further evaluation of the relationships between weight change and circulating lipids in patients with IPF is required.

A study in patients with chronic obstructive pulmonary disease demonstrated inverse relationships between individual sphingolipids and the severity of emphysema, and measurements of select sphingolipids improved prediction of severe exacerbations beyond predictions based on demographic and clinical covariates [36]. In studies in patients with IPF, negative correlations were observed between levels of select signaling lipids in peripheral blood and lung function [34], and higher levels of certain triglycerides and phosphatidylcholines were found in the plasma of patients with more rapid progression [21]. A risk model based on expression of select fatty acid genes in bronchoalveolar lavage fluid predicted outcomes among patients with IPF [33]. In our study, higher levels of ceramide C18:1/C16:0 was associated with more severe disease. Interestingly, this ceramide has been associated with inhibition of mitochondrial fatty acid oxidation [37], which may support the acylcarnitine profile described in our study in the sense that limits of mitochondrial oxidative activity could lead to accumulation of partially oxidized fatty acyl CoAs and acylcarnitines [38]. Of note, this particular ceramide has been linked to defects in mitochondrial fragmentation and a plethora of metabolic disease phenotypes [39], supporting the concept of IPF as a systemic metabolic disorder.

We found evidence of amino acid dysregulation in patients with IPF. Specifically, patients with IPF had significantly higher circulating levels of ornithine compared with controls. Ornithine, a non-proteinogenic amino acid, is derived from the actions of arginase and serves as an important intermediate in the urea cycle and as a precursor to other metabolites, including citrulline, proline and various polyamines important for wound healing and cell proliferation [40]. Of note, proline and its derivative hydroxyproline comprise approximately 23% of the amino acid content of collagen [41], suggesting that higher levels of ornithine in the blood may be reflective of enhanced collagen production in the lung. A previous study reported elevations in 4-hydroxyproline levels and of the polyamines putrescine and spermidine in the lungs of patients with IPF [42], supporting the concept that ornithine metabolism is dysregulated in this disease. Our study also indicated that higher levels of certain amino acids were correlated with less severe disease: higher levels of the branched chain amino acids valine, leucine and isoleucine were associated with higher DLco % predicted and lower CPI. In addition to being key substrates for energy metabolism and protein synthesis, these amino acids play key signaling roles in regulating growth and energy pathways. Indeed, they are major activators of the mammalian target of rapamycin (mTOR), a pro-growth enzyme that is hyperactivated in fibroblasts and epithelial cells in IPF [43–45]. Whether higher circulating levels of these amino acids correlate with mTOR activity in the lung is a concept worthy of further investigation.

Our study has several strengths, including the multicenter nature of the registries from which the IPF and control cohorts were derived and the comparison of patients with IPF to control participants with similar distributions of age, sex, and smoking history. There are also some inherent limitations in our approach. Our cohort is a US-based population of predominantly White patients and its generalizability to other populations of patients with IPF is uncertain. Although we characterized a broad array of metabolites, our approach was targeted rather than discovery-based, so metabolites of potential importance may have been missed. The analyses of clinical analytes did not include all the participants in the study due to insufficient sample volumes.

## Conclusion

The results of this study suggest that circulating metabolites may hold value as diagnostic, disease activity, or prognostic biomarkers for IPF and may provide insights into the pathobiology of this disease. These data provide support for the development of biomarker-inclusive algorithms that enable risk stratification for patients with IPF. Multicenter cohort studies such as the IPF-PRO Registry, with well-annotated serial clinical data and biological samples, are of great value for identification and validation of biomarkers to improve the management of IPF.

A podcast describing the key data presented in this manuscript is available at: https://www.usscicomms.com/respiratory/Todd/IPF-PRO-BIOmetabolomics.

### Electronic supplementary material

Below is the link to the electronic supplementary material.

**Additional file 1: Table S1.** Concentrations of metabolites in the IPF and control cohorts; **Table S2.** Concentrations of clinical analytes in the IPF and control cohorts; **Table S3.** Differences in metabolite levels between IPF cases and controls; **Table S4.** Differences in clinical analyte levels between the IPF and control cohorts; **Table S5.** Differences in metabolite and clinical analyte levels between patients with IPF who were and were not using anti-fibrotic therapy at enrollment; **Table S6.** Associations of circulating metabolites with DLco % predicted at enrollment in patients with IPF, unadjusted and adjusted for use of anti-fibrotic therapy at enrollment; **Table S7.** Association of circulating metabolites with FVC % predicted at enrollment in patients with IPF, unadjusted and adjusted for use of anti-fibrotic therapy at enrollment; **Table S8.** Association of circulating metabolites with composite physiologic index at enrollment in patients with IPF unadjusted and adjusted for use of anti-fibrotic therapy at enrollment; **Figure S1.** Adjusted univariate associations between metabolites and risk of outcomes

## Data Availability

The datasets analyzed during the current study are not publicly available, but they are available from the corresponding author on reasonable request.

## Abbreviations

BMI: Body mass index
CPI: Composite physiologic index
DLco: Diffusion capacity of the lung for carbon monoxide
FDR: False discovery rate
FEV_1_: Forced expiratory volume in 1 s
FVC: Forced vital capacity
HDL: High-density lipoprotein
IPF: Idiopathic pulmonary fibrosis
LC: Liquid chromatography
LDL: Low-density lipoprotein
MS/MS: Mass spectrometry
mTOR: Mammalian target of rapamycin
NEFA: Non-esterified fatty acids
