# Supplementary material for: Circulating metabolic profile in idiopathic pulmonary fibrosis: data from the IPF-PRO Registry
Source: Respir Res. 2024 Jan 25;25:58. doi: 10.1186/s12931-023-02644-7 (PMC10809477; doi:10.1186/s12931-023-02644-7)

**Circulating Metabolic Profile in Idiopathic Pulmonary Fibrosis: Data from the IPF-PRO Registry (Summer R et al)**

**SUPPLEMENTARY MATERIAL**

**Additional file 1**

**Table S1.** Concentrations of metabolites in the IPF and control cohorts.

| **Metabolite** | **Cohort** | **Metabolite concentration** | | | | |
| --- | --- | --- | --- | --- | --- | --- |
|  |  | **Mean** | **Standard deviation** | **25^th^ percentile** | **Median** | **75^th^ percentile** |
| **Acylcarnitines** |  |  |  |  |  |  |
| C2 | Control | 7.180 | 2.591 | 5.317 | 6.673 | 8.276 |
|  | IPF | 7.916 | 3.149 | 5.647 | 7.216 | 9.573 |
| C3 | Control | 0.407 | 0.140 | 0.312 | 0.370 | 0.482 |
|  | IPF | 0.419 | 0.193 | 0.296 | 0.388 | 0.501 |
| C4/Ci4 | Control | 0.222 | 0.096 | 0.157 | 0.204 | 0.269 |
|  | IPF | 0.237 | 0.130 | 0.154 | 0.210 | 0.283 |
| C5:1 | Control | 0.063 | 0.016 | 0.051 | 0.064 | 0.075 |
|  | IPF | 0.067 | 0.016 | 0.056 | 0.065 | 0.075 |
| C5 | Control | 0.163 | 0.083 | 0.101 | 0.146 | 0.198 |
|  | IPF | 0.158 | 0.080 | 0.104 | 0.145 | 0.193 |
| C4-OH | Control | 0.043 | 0.028 | 0.024 | 0.038 | 0.056 |
|  | IPF | 0.051 | 0.038 | 0.027 | 0.039 | 0.063 |
| C6 | Control | 0.070 | 0.037 | 0.047 | 0.063 | 0.081 |
|  | IPF | 0.085 | 0.073 | 0.058 | 0.075 | 0.096 |
| C5-OH/C3-DC | Control | 0.033 | 0.012 | 0.024 | 0.030 | 0.039 |
|  | IPF | 0.038 | 0.020 | 0.027 | 0.035 | 0.045 |
| C4-DC/Ci4-DC | Control | 0.041 | 0.012 | 0.033 | 0.037 | 0.048 |
|  | IPF | 0.048 | 0.031 | 0.036 | 0.044 | 0.052 |
| C8:1 | Control | 0.326 | 0.174 | 0.197 | 0.316 | 0.413 |
|  | IPF | 0.302 | 0.151 | 0.195 | 0.272 | 0.376 |
| C8 | Control | 0.115 | 0.083 | 0.067 | 0.096 | 0.128 |
|  | IPF | 0.158 | 0.255 | 0.089 | 0.123 | 0.165 |
| C5-DC | Control | 0.055 | 0.020 | 0.041 | 0.053 | 0.068 |
|  | IPF | 0.059 | 0.029 | 0.044 | 0.054 | 0.067 |
| C8:1-OH/C6:1-DC | Control | 0.031 | 0.012 | 0.023 | 0.029 | 0.036 |
|  | IPF | 0.034 | 0.015 | 0.023 | 0.031 | 0.040 |
| C6-DC/C8-OH | Control | 0.068 | 0.033 | 0.045 | 0.059 | 0.083 |
|  | IPF | 0.080 | 0.044 | 0.053 | 0.072 | 0.092 |
| C10:3 | Control | 0.091 | 0.050 | 0.056 | 0.080 | 0.107 |
|  | IPF | 0.090 | 0.050 | 0.054 | 0.082 | 0.111 |
| C10:2 | Control | 0.030 | 0.018 | 0.020 | 0.026 | 0.034 |
|  | IPF | 0.029 | 0.014 | 0.019 | 0.026 | 0.034 |
| C10:1 | Control | 0.141 | 0.088 | 0.093 | 0.131 | 0.160 |
|  | IPF | 0.157 | 0.076 | 0.110 | 0.142 | 0.193 |
| C10 | Control | 0.162 | 0.115 | 0.090 | 0.141 | 0.179 |
|  | IPF | 0.227 | 0.345 | 0.122 | 0.176 | 0.243 |
| C7-DC | Control | 0.013 | 0.007 | 0.009 | 0.013 | 0.017 |
|  | IPF | 0.015 | 0.009 | 0.009 | 0.014 | 0.019 |
| C8:1-DC | Control | 0.029 | 0.012 | 0.020 | 0.026 | 0.034 |
|  | IPF | 0.034 | 0.017 | 0.024 | 0.032 | 0.041 |
| C10-OH/C8-DC | Control | 0.040 | 0.018 | 0.028 | 0.034 | 0.047 |
|  | IPF | 0.050 | 0.022 | 0.034 | 0.047 | 0.060 |
| C12:1 | Control | 0.104 | 0.053 | 0.070 | 0.095 | 0.129 |
|  | IPF | 0.121 | 0.057 | 0.085 | 0.110 | 0.149 |
| C12 | Control | 0.063 | 0.033 | 0.043 | 0.056 | 0.075 |
|  | IPF | 0.085 | 0.081 | 0.054 | 0.072 | 0.097 |
| C12-OH/C10-DC | Control | 0.011 | 0.004 | 0.007 | 0.010 | 0.013 |
|  | IPF | 0.013 | 0.007 | 0.009 | 0.012 | 0.016 |
| C14:2 | Control | 0.038 | 0.021 | 0.023 | 0.035 | 0.046 |
|  | IPF | 0.047 | 0.027 | 0.029 | 0.041 | 0.059 |
| C14:1 | Control | 0.057 | 0.029 | 0.036 | 0.049 | 0.072 |
|  | IPF | 0.077 | 0.060 | 0.046 | 0.063 | 0.092 |
| C14 | Control | 0.024 | 0.007 | 0.019 | 0.023 | 0.030 |
|  | IPF | 0.034 | 0.017 | 0.025 | 0.031 | 0.038 |
| C14:1-OH | Control | 0.014 | 0.005 | 0.010 | 0.013 | 0.016 |
|  | IPF | 0.016 | 0.006 | 0.012 | 0.015 | 0.020 |
| C14-OH/C12-DC | Control | 0.009 | 0.004 | 0.006 | 0.008 | 0.011 |
|  | IPF | 0.010 | 0.004 | 0.007 | 0.009 | 0.012 |
| C16:2 | Control | 0.009 | 0.004 | 0.006 | 0.009 | 0.011 |
|  | IPF | 0.012 | 0.009 | 0.007 | 0.009 | 0.014 |
| C16:1 | Control | 0.019 | 0.009 | 0.013 | 0.018 | 0.024 |
|  | IPF | 0.029 | 0.021 | 0.018 | 0.025 | 0.034 |
| C16 | Control | 0.073 | 0.017 | 0.061 | 0.073 | 0.083 |
|  | IPF | 0.105 | 0.065 | 0.084 | 0.097 | 0.117 |
| C16:1-OH/C14:1-DC | Control | 0.007 | 0.003 | 0.005 | 0.006 | 0.009 |
|  | IPF | 0.009 | 0.004 | 0.006 | 0.008 | 0.011 |
| C16-OH/C14-DC | Control | 0.006 | 0.003 | 0.004 | 0.005 | 0.007 |
|  | IPF | 0.006 | 0.003 | 0.004 | 0.006 | 0.008 |
| C18:2 | Control | 0.057 | 0.020 | 0.043 | 0.054 | 0.069 |
|  | IPF | 0.091 | 0.040 | 0.066 | 0.084 | 0.104 |
| C18:1 | Control | 0.097 | 0.032 | 0.073 | 0.093 | 0.120 |
|  | IPF | 0.154 | 0.086 | 0.115 | 0.145 | 0.178 |
| C18 | Control | 0.044 | 0.009 | 0.037 | 0.043 | 0.049 |
|  | IPF | 0.059 | 0.042 | 0.046 | 0.054 | 0.064 |
| C18:2-OH | Control | 0.006 | 0.002 | 0.005 | 0.006 | 0.007 |
|  | IPF | 0.006 | 0.002 | 0.005 | 0.006 | 0.008 |
| C18:1-OH/C16:1-DC | Control | 0.006 | 0.002 | 0.004 | 0.006 | 0.007 |
|  | IPF | 0.007 | 0.003 | 0.004 | 0.006 | 0.008 |
| C18-OH/C16-DC | Control | 0.007 | 0.004 | 0.005 | 0.007 | 0.009 |
|  | IPF | 0.009 | 0.004 | 0.006 | 0.008 | 0.011 |
| C20:4 | Control | 0.005 | 0.002 | 0.004 | 0.005 | 0.006 |
|  | IPF | 0.008 | 0.003 | 0.005 | 0.007 | 0.009 |
| C20 | Control | 0.004 | 0.002 | 0.002 | 0.004 | 0.005 |
|  | IPF | 0.004 | 0.002 | 0.003 | 0.004 | 0.006 |
| C18:1-DC | Control | 0.008 | 0.003 | 0.006 | 0.008 | 0.010 |
|  | IPF | 0.010 | 0.006 | 0.007 | 0.009 | 0.012 |
| C20-OH/C18-DC | Control | 0.009 | 0.004 | 0.006 | 0.008 | 0.011 |
|  | IPF | 0.009 | 0.007 | 0.007 | 0.008 | 0.011 |
| C22 | Control | 0.005 | 0.002 | 0.003 | 0.005 | 0.006 |
|  | IPF | 0.004 | 0.002 | 0.003 | 0.004 | 0.005 |
| **Amino acids** |  |  |  |  |  |  |
| Gly | Control | 295.634 | 63.698 | 251.017 | 290.281 | 327.666 |
|  | IPF | 295.700 | 67.808 | 256.255 | 285.664 | 322.231 |
| Ala | Control | 456.411 | 115.529 | 367.879 | 433.485 | 530.673 |
|  | IPF | 453.302 | 107.685 | 382.488 | 434.780 | 505.257 |
| Ser | Control | 91.875 | 19.528 | 75.979 | 91.412 | 106.284 |
|  | IPF | 100.551 | 26.490 | 84.117 | 97.402 | 112.324 |
| Pro | Control | 238.568 | 78.829 | 184.265 | 223.967 | 282.778 |
|  | IPF | 235.728 | 66.766 | 194.495 | 223.930 | 271.648 |
| Val | Control | 239.028 | 57.136 | 199.240 | 226.045 | 277.338 |
|  | IPF | 238.596 | 50.979 | 202.745 | 233.861 | 271.743 |
| Leu/Ile | Control | 174.486 | 52.554 | 134.628 | 163.839 | 206.003 |
|  | IPF | 174.525 | 43.089 | 142.396 | 169.578 | 198.500 |
| Met | Control | 28.704 | 6.712 | 24.038 | 27.564 | 32.246 |
|  | IPF | 27.905 | 7.049 | 22.507 | 26.966 | 31.543 |
| His | Control | 78.174 | 11.455 | 70.943 | 77.268 | 84.234 |
|  | IPF | 76.980 | 12.798 | 68.358 | 76.319 | 84.524 |
| Phe | Control | 64.165 | 11.502 | 55.531 | 62.625 | 70.245 |
|  | IPF | 68.245 | 12.590 | 59.380 | 66.758 | 76.030 |
| Tyr | Control | 69.878 | 15.753 | 58.117 | 67.809 | 78.068 |
|  | IPF | 77.154 | 18.642 | 63.305 | 75.547 | 88.744 |
| Asx | Control | 67.888 | 28.473 | 50.558 | 60.074 | 73.324 |
|  | IPF | 65.657 | 18.187 | 53.786 | 63.388 | 72.270 |
| Glx | Control | 112.584 | 29.609 | 93.845 | 109.202 | 123.341 |
|  | IPF | 120.294 | 32.555 | 95.905 | 115.203 | 137.738 |
| Orn | Control | 67.858 | 12.606 | 59.295 | 66.747 | 75.306 |
|  | IPF | 95.416 | 27.009 | 75.196 | 92.259 | 112.641 |
| Cit | Control | 34.922 | 11.061 | 27.943 | 34.371 | 41.830 |
|  | IPF | 37.943 | 11.703 | 30.799 | 37.147 | 45.459 |
| Arg | Control | 83.210 | 17.138 | 73.009 | 80.458 | 92.272 |
|  | IPF | 70.200 | 21.267 | 53.767 | 69.843 | 83.186 |
| **Ceramides** |  |  |  |  |  |  |
| Cer(d18:1/14:0) | Control | 0.009 | 0.003 | 0.006 | 0.008 | 0.011 |
|  | IPF | 0.011 | 0.004 | 0.008 | 0.010 | 0.013 |
| Cer(d18:1/16:0) | Control | 0.290 | 0.068 | 0.240 | 0.283 | 0.337 |
|  | IPF | 0.363 | 0.099 | 0.289 | 0.351 | 0.421 |
| Cer(d18:1/18:0) | Control | 0.102 | 0.034 | 0.076 | 0.098 | 0.124 |
|  | IPF | 0.133 | 0.045 | 0.099 | 0.127 | 0.157 |
| Cer(d18:1/20:1) | Control | 0.007 | 0.003 | 0.005 | 0.006 | 0.008 |
|  | IPF | 0.008 | 0.003 | 0.006 | 0.008 | 0.010 |
| Cer(d18:1/20:0) | Control | 0.109 | 0.058 | 0.068 | 0.092 | 0.137 |
|  | IPF | 0.148 | 0.090 | 0.093 | 0.127 | 0.173 |
| Cer(d18:1/22:0) | Control | 0.665 | 0.202 | 0.516 | 0.656 | 0.760 |
|  | IPF | 0.733 | 0.224 | 0.574 | 0.687 | 0.841 |
| Cer(d18:1/23:0) | Control | 0.498 | 0.160 | 0.388 | 0.480 | 0.579 |
|  | IPF | 0.555 | 0.171 | 0.447 | 0.521 | 0.633 |
| Cer(d18:1/24:1) | Control | 0.740 | 0.197 | 0.598 | 0.736 | 0.867 |
|  | IPF | 0.954 | 0.304 | 0.748 | 0.912 | 1.100 |
| Cer(d18:1/24:0) | Control | 2.020 | 0.580 | 1.604 | 2.011 | 2.313 |
|  | IPF | 2.153 | 0.635 | 1.709 | 2.037 | 2.535 |
| Cer(d18:1/25:0) | Control | 0.198 | 0.067 | 0.140 | 0.194 | 0.236 |
|  | IPF | 0.239 | 0.085 | 0.178 | 0.227 | 0.283 |
| Cer(d18:1/26:1) | Control | 0.086 | 0.051 | 0.048 | 0.075 | 0.113 |
|  | IPF | 0.120 | 0.068 | 0.069 | 0.103 | 0.154 |
| Cer(d18:1/26:0) | Control | 0.241 | 0.169 | 0.132 | 0.197 | 0.301 |
|  | IPF | 0.292 | 0.192 | 0.152 | 0.243 | 0.391 |
| GlcCer(d18:1/16:0) | Control | 0.152 | 0.044 | 0.118 | 0.153 | 0.179 |
|  | IPF | 0.197 | 0.068 | 0.148 | 0.182 | 0.235 |
| GlcCer(d18:1/18:0) | Control | 0.033 | 0.011 | 0.025 | 0.033 | 0.041 |
|  | IPF | 0.041 | 0.015 | 0.031 | 0.038 | 0.048 |
| GlcCer(d18:1/20:0) | Control | 0.028 | 0.010 | 0.021 | 0.027 | 0.034 |
|  | IPF | 0.034 | 0.014 | 0.025 | 0.031 | 0.041 |
| GlcCer(d18:1/22:0) | Control | 0.223 | 0.081 | 0.165 | 0.217 | 0.275 |
|  | IPF | 0.240 | 0.090 | 0.172 | 0.223 | 0.295 |
| GlcCer(d18:1/23:0) | Control | 0.116 | 0.044 | 0.085 | 0.106 | 0.142 |
|  | IPF | 0.132 | 0.054 | 0.092 | 0.120 | 0.160 |
| GlcCer(d18:1/24:1) | Control | 0.219 | 0.069 | 0.159 | 0.215 | 0.267 |
|  | IPF | 0.275 | 0.100 | 0.200 | 0.254 | 0.327 |
| GlcCer(d18:1/24:0) | Control | 0.328 | 0.110 | 0.253 | 0.315 | 0.402 |
|  | IPF | 0.364 | 0.130 | 0.270 | 0.348 | 0.447 |
| GlcCer(d18:1/26:1) | Control | 0.007 | 0.005 | 0.003 | 0.006 | 0.009 |
|  | IPF | 0.009 | 0.007 | 0.004 | 0.008 | 0.012 |
| GlcCer(d18:1/26:0) | Control | 0.015 | 0.010 | 0.007 | 0.011 | 0.020 |
|  | IPF | 0.019 | 0.015 | 0.010 | 0.016 | 0.023 |
| **Sphingomyelins** |  |  |  |  |  |  |
| SM(d31:1) | Control | 0.072 | 0.021 | 0.057 | 0.070 | 0.082 |
|  | IPF | 0.077 | 0.023 | 0.060 | 0.074 | 0.091 |
| SM(d31:0) | Control | 0.012 | 0.003 | 0.009 | 0.011 | 0.013 |
|  | IPF | 0.013 | 0.004 | 0.010 | 0.012 | 0.014 |
| SM(d32:1) | Control | 2.156 | 0.564 | 1.688 | 2.128 | 2.534 |
|  | IPF | 2.414 | 0.666 | 1.960 | 2.343 | 2.822 |
| SM(d33:1) | Control | 1.124 | 0.282 | 0.935 | 1.067 | 1.268 |
|  | IPF | 1.306 | 0.342 | 1.070 | 1.267 | 1.502 |
| SM(d34:2) | Control | 4.144 | 0.945 | 3.477 | 4.018 | 4.812 |
|  | IPF | 4.216 | 0.973 | 3.545 | 4.104 | 4.802 |
| SM(d34:1) | Control | 21.147 | 3.905 | 18.259 | 20.653 | 24.393 |
|  | IPF | 23.010 | 4.314 | 19.982 | 22.369 | 25.822 |
| SM(d35:3) | Control | 0.295 | 0.465 | 0.049 | 0.126 | 0.389 |
|  | IPF | 0.442 | 0.696 | 0.057 | 0.161 | 0.545 |
| SM(d35:1) | Control | 0.721 | 0.162 | 0.611 | 0.701 | 0.828 |
|  | IPF | 0.812 | 0.205 | 0.664 | 0.799 | 0.930 |
| SM(d36:2) | Control | 2.883 | 0.863 | 2.304 | 2.739 | 3.278 |
|  | IPF | 2.879 | 0.787 | 2.275 | 2.785 | 3.336 |
| SM(d37:2) | Control | 0.165 | 0.056 | 0.128 | 0.153 | 0.188 |
|  | IPF | 0.174 | 0.061 | 0.133 | 0.163 | 0.203 |
| SM(d37:1) | Control | 0.497 | 0.146 | 0.386 | 0.479 | 0.581 |
|  | IPF | 0.550 | 0.166 | 0.439 | 0.532 | 0.624 |
| SM(d38:2) | Control | 1.731 | 0.436 | 1.419 | 1.659 | 1.932 |
|  | IPF | 1.741 | 0.420 | 1.420 | 1.690 | 2.000 |
| SM(d38:1) | Control | 4.199 | 0.981 | 3.334 | 4.144 | 4.856 |
|  | IPF | 4.327 | 0.980 | 3.628 | 4.231 | 4.936 |
| SM(d39:2) | Control | 0.309 | 0.092 | 0.241 | 0.289 | 0.359 |
|  | IPF | 0.311 | 0.088 | 0.249 | 0.302 | 0.366 |
| SM(d40:2) | Control | 7.071 | 1.643 | 5.949 | 6.830 | 8.054 |
|  | IPF | 6.883 | 1.567 | 5.650 | 6.647 | 7.847 |
| SM(d40:1) | Control | 9.188 | 2.308 | 7.469 | 9.148 | 10.713 |
|  | IPF | 8.966 | 2.211 | 7.374 | 8.661 | 10.395 |
| SM(d41:2) | Control | 2.781 | 0.731 | 2.195 | 2.673 | 3.191 |
|  | IPF | 2.800 | 0.669 | 2.354 | 2.701 | 3.164 |
| SM(d41:1) | Control | 3.725 | 0.934 | 3.010 | 3.659 | 4.374 |
|  | IPF | 3.692 | 0.830 | 3.119 | 3.605 | 4.230 |
| SM(d42:3) | Control | 7.944 | 2.360 | 6.130 | 7.594 | 9.183 |
|  | IPF | 8.437 | 2.204 | 6.869 | 8.148 | 9.747 |
| SM(d42:2) | Control | 15.276 | 3.594 | 12.402 | 14.785 | 17.383 |
|  | IPF | 16.454 | 3.749 | 13.873 | 15.748 | 18.710 |
| SM(d42:1) | Control | 7.613 | 1.837 | 6.220 | 7.564 | 8.724 |
|  | IPF | 7.597 | 2.112 | 6.245 | 7.334 | 8.692 |
| SM(d43:3) | Control | 0.206 | 0.114 | 0.129 | 0.167 | 0.237 |
|  | IPF | 0.241 | 0.143 | 0.155 | 0.199 | 0.264 |
| SM(d43:2) | Control | 0.660 | 0.202 | 0.532 | 0.608 | 0.765 |
|  | IPF | 0.776 | 0.215 | 0.635 | 0.757 | 0.911 |
| SM(d43:1) | Control | 0.528 | 0.134 | 0.442 | 0.508 | 0.590 |
|  | IPF | 0.553 | 0.137 | 0.461 | 0.535 | 0.633 |
| SM(d44:2) | Control | 0.452 | 0.285 | 0.297 | 0.364 | 0.501 |
|  | IPF | 0.480 | 0.325 | 0.304 | 0.400 | 0.552 |
| SM(d44:1) | Control | 0.228 | 0.138 | 0.146 | 0.192 | 0.256 |
|  | IPF | 0.235 | 0.162 | 0.155 | 0.196 | 0.268 |
| SM(d44:0) | Control | 0.052 | 0.019 | 0.038 | 0.047 | 0.063 |
|  | IPF | 0.055 | 0.023 | 0.040 | 0.051 | 0.064 |
| SM(d45:1) | Control | 0.039 | 0.033 | 0.023 | 0.030 | 0.045 |
|  | IPF | 0.039 | 0.021 | 0.025 | 0.033 | 0.048 |
| SM(d45:0) | Control | 0.064 | 0.042 | 0.036 | 0.051 | 0.080 |
|  | IPF | 0.065 | 0.047 | 0.036 | 0.050 | 0.078 |
| SM(d33:3) | Control | 0.473 | 0.755 | 0.100 | 0.216 | 0.593 |
|  | IPF | 0.682 | 1.004 | 0.121 | 0.285 | 0.828 |
| SM(d36:1) | Control | 5.097 | 1.359 | 4.197 | 4.781 | 6.020 |
|  | IPF | 5.548 | 1.398 | 4.545 | 5.348 | 6.318 |
| SM(d39:3) | Control | 0.211 | 0.296 | 0.067 | 0.108 | 0.239 |
|  | IPF | 0.266 | 0.361 | 0.067 | 0.124 | 0.329 |
| SM(d39:1) | Control | 1.522 | 0.436 | 1.174 | 1.481 | 1.821 |
|  | IPF | 1.536 | 0.409 | 1.246 | 1.485 | 1.776 |
| SM(d40:3) | Control | 0.895 | 0.409 | 0.594 | 0.771 | 1.058 |
|  | IPF | 0.964 | 0.416 | 0.680 | 0.867 | 1.141 |
| **Keto-acids** |  |  |  |  |  |  |
| KIV | Control | 12.275 | 2.399 | 10.540 | 12.411 | 13.683 |
|  | IPF | 12.456 | 2.796 | 10.539 | 12.170 | 14.182 |
| KIC | Control | 32.266 | 8.083 | 26.911 | 30.551 | 37.580 |
|  | IPF | 29.590 | 9.016 | 23.225 | 28.892 | 34.328 |
| KMV | Control | 20.874 | 6.028 | 16.859 | 19.810 | 24.218 |
|  | IPF | 20.160 | 6.011 | 15.854 | 19.561 | 23.905 |
| **Other** |  |  |  |  |  |  |
| 3-HIB | Control | 15.697 | 7.960 | 9.942 | 13.186 | 19.126 |
|  | IPF | 14.918 | 6.264 | 10.764 | 13.570 | 17.858 |

No patients had missing data for any metabolite.

**Table S2.** Concentrations of clinical analytes in the IPF and control cohorts.

| **Clinical analyte** | **Cohort** | **% with missing data** | **Concentration** | | | | |
| --- | --- | --- | --- | --- | --- | --- | --- |
|  |  |  | **Mean** | **Standard deviation** | **25^th^ percentile** | **Median** | **75^th^ percentile** |
| Cholesterol | Control | 0 | 170.730 | 44.253 | 140.000 | 165.500 | 197.000 |
|  | IPF | 0 | 200.490 | 46.447 | 167.000 | 200.000 | 224.000 |
| Glucose | Control | 0 | 122.460 | 58.226 | 94.000 | 103.500 | 127.500 |
|  | IPF | 0 | 108.800 | 33.311 | 90.000 | 100.500 | 118.000 |
| Ketones | Control | 0 | 100.667 | 90.173 | 38.200 | 65.600 | 138.800 |
|  | IPF | 0.3 | 127.918 | 138.759 | 52.300 | 84.000 | 140.200 |
| Lactate | Control | 0 | 1.989 | 1.068 | 1.300 | 1.700 | 2.400 |
|  | IPF | 0 | 2.037 | 0.808 | 1.500 | 1.900 | 2.400 |
| NEFA | Control | 0 | 0.321 | 0.225 | 0.160 | 0.260 | 0.415 |
|  | IPF | 0 | 0.518 | 0.297 | 0.300 | 0.470 | 0.690 |
| TG | Control | 0 | 174.570 | 102.919 | 99.500 | 148.000 | 219.500 |
|  | IPF | 0 | 154.083 | 83.904 | 97.000 | 132.500 | 191.000 |
| Glycerol | Control | 0 | 0.587 | 0.284 | 0.395 | 0.535 | 0.695 |
|  | IPF | 0 | 0.736 | 0.464 | 0.470 | 0.640 | 0.840 |
| 3-HB | Control | 0 | 69.551 | 68.511 | 23.550 | 41.100 | 97.750 |
|  | IPF | 12.3 | 86.154 | 102.807 | 30.100 | 55.100 | 96.600 |
| HDL | Control | 0 | 57.408 | 16.080 | 44.400 | 56.350 | 66.550 |
|  | IPF | 5.3 | 52.356 | 15.545 | 40.400 | 48.850 | 60.950 |
| LDL | Control | 0 | 107.242 | 37.939 | 79.550 | 97.600 | 130.300 |
|  | IPF | 1.3 | 100.252 | 31.026 | 77.850 | 95.500 | 119.650 |

**Table S3.** Differences in metabolite levels between IPF cases and controls.

| **Metabolite** | **IPF cohort  (mean)** | **Control cohort (mean)** | **Log_2_-fold change (IPF vs control)** | **Raw *P* value** | **FDR-corrected *P* value** |
| --- | --- | --- | --- | --- | --- |
| **Acylcarnitines** |  |  |  |  |  |
| C2 | 7.916 | 7.180 | 0.123 | 0.040 | 0.069 |
| C3 | 0.419 | 0.407 | -0.004 | 0.951 | 0.978 |
| C4/Ci4 | 0.237 | 0.222 | 0.039 | 0.609 | 0.747 |
| C5:1 | 0.067 | 0.063 | 0.101 | 0.013 | 0.026 |
| C5 | 0.158 | 0.163 | -0.052 | 0.491 | 0.615 |
| C4-OH | 0.051 | 0.043 | 0.187 | 0.086 | 0.136 |
| C6 | 0.085 | 0.070 | 0.278 | 0.005 | 0.012 |
| C5-OH/C3-DC | 0.038 | 0.033 | 0.169 | 0.007 | 0.015 |
| C4-DC/Ci4-DC | 0.048 | 0.041 | 0.171 | 0.002 | 0.007 |
| C8:1 | 0.302 | 0.326 | -0.090 | 0.275 | 0.368 |
| C8 | 0.158 | 0.115 | 0.354 | < 0.001 | < 0.001 |
| C5-DC | 0.059 | 0.055 | 0.066 | 0.323 | 0.418 |
| C8:1-OH/C6:1-DC | 0.034 | 0.031 | 0.093 | 0.168 | 0.256 |
| C6-DC/C8-OH | 0.080 | 0.068 | 0.226 | 0.003 | 0.008 |
| C10:3 | 0.090 | 0.091 | -0.018 | 0.838 | 0.908 |
| C10:2 | 0.029 | 0.030 | -0.039 | 0.628 | 0.762 |
| C10:1 | 0.157 | 0.141 | 0.181 | 0.024 | 0.045 |
| C10 | 0.227 | 0.162 | 0.389 | < 0.001 | < 0.001 |
| C7-DC | 0.015 | 0.013 | 0.162 | 0.254 | 0.351 |
| C8:1-DC | 0.034 | 0.029 | 0.220 | 0.003 | 0.008 |
| C10-OH/C8-DC | 0.050 | 0.040 | 0.351 | < 0.001 | < 0.001 |
| C12:1 | 0.121 | 0.104 | 0.250 | 0.001 | 0.003 |
| C12 | 0.085 | 0.063 | 0.362 | < 0.001 | < 0.001 |
| C12-OH/C10-DC | 0.013 | 0.011 | 0.277 | < 0.001 | < 0.001 |
| C14:2 | 0.047 | 0.038 | 0.297 | < 0.001 | 0.003 |
| C14:1 | 0.077 | 0.057 | 0.389 | < 0.001 | < 0.001 |
| C14 | 0.034 | 0.024 | 0.423 | < 0.001 | < 0.001 |
| C14:1-OH | 0.016 | 0.014 | 0.241 | < 0.001 | < 0.001 |
| C14-OH/C12-DC | 0.010 | 0.009 | 0.159 | 0.029 | 0.053 |
| C16:2 | 0.012 | 0.009 | 0.304 | < 0.001 | 0.003 |
| C16:1 | 0.029 | 0.019 | 0.528 | < 0.001 | < 0.001 |
| C16 | 0.105 | 0.073 | 0.478 | < 0.001 | < 0.001 |
| C16:1-OH/C14:1-DC | 0.009 | 0.007 | 0.369 | < 0.001 | < 0.001 |
| C16-OH/C14-DC | 0.006 | 0.006 | 0.150 | 0.056 | 0.092 |
| C18:2 | 0.091 | 0.057 | 0.654 | < 0.001 | < 0.001 |
| C18:1 | 0.154 | 0.097 | 0.649 | < 0.001 | < 0.001 |
| C18 | 0.059 | 0.044 | 0.361 | < 0.001 | < 0.001 |
| C18:2-OH | 0.006 | 0.006 | 0.025 | 0.741 | 0.831 |
| C18:1-OH/C16:1-DC | 0.007 | 0.006 | 0.102 | 0.268 | 0.362 |
| C18-OH/C16-DC | 0.009 | 0.007 | 0.291 | < 0.001 | < 0.001 |
| C20:4 | 0.008 | 0.005 | 0.621 | < 0.001 | < 0.001 |
| C20 | 0.004 | 0.004 | 0.176 | 0.177 | 0.266 |
| C18:1-DC | 0.010 | 0.008 | 0.201 | 0.016 | 0.031 |
| C20-OH/C18-DC | 0.009 | 0.009 | 0.030 | 0.732 | 0.830 |
| C22 | 0.004 | 0.005 | -0.384 | 0.003 | 0.007 |
| **Amino Acids** |  |  |  |  |  |
| Gly | 295.700 | 295.634 | -0.001 | 0.982 | 0.990 |
| Ala | 453.302 | 456.411 | -0.002 | 0.953 | 0.978 |
| Ser | 100.551 | 91.875 | 0.122 | 0.002 | 0.005 |
| Pro | 235.728 | 238.568 | -0.002 | 0.969 | 0.986 |
| Val | 238.596 | 239.028 | 0.004 | 0.902 | 0.950 |
| Leu/Ile | 174.525 | 174.486 | 0.016 | 0.711 | 0.813 |
| Met | 27.905 | 28.704 | -0.049 | 0.215 | 0.311 |
| His | 76.980 | 78.174 | -0.027 | 0.314 | 0.416 |
| Phe | 68.245 | 64.165 | 0.087 | 0.003 | 0.008 |
| Tyr | 77.154 | 69.878 | 0.136 | < 0.001 | 0.002 |
| Asx | 65.657 | 67.888 | -0.000 | 0.996 | 0.996 |
| Glx | 120.294 | 112.584 | 0.090 | 0.035 | 0.062 |
| Orn | 95.416 | 67.858 | 0.459 | < 0.001 | < 0.001 |
| Cit | 37.943 | 34.922 | 0.122 | 0.038 | 0.068 |
| Arg | 70.200 | 83.210 | -0.289 | < 0.001 | < 0.001 |
| **Ceramides** |  |  |  |  |  |
| Cer(d18:1/14:0) | 0.011 | 0.009 | 0.291 | < 0.001 | < 0.001 |
| Cer(d18:1/16:0) | 0.363 | 0.290 | 0.312 | < 0.001 | < 0.001 |
| Cer(d18:1/18:0) | 0.133 | 0.102 | 0.385 | < 0.001 | < 0.001 |
| Cer(d18:1/20:1) | 0.008 | 0.007 | 0.273 | < 0.001 | < 0.001 |
| Cer(d18:1/20:0) | 0.148 | 0.109 | 0.422 | < 0.001 | < 0.001 |
| Cer(d18:1/22:0) | 0.733 | 0.665 | 0.145 | 0.003 | 0.008 |
| Cer(d18:1/23:0) | 0.555 | 0.498 | 0.165 | < 0.001 | 0.003 |
| Cer(d18:1/24:1) | 0.954 | 0.740 | 0.356 | < 0.001 | < 0.001 |
| Cer(d18:1/24:0) | 2.153 | 2.020 | 0.093 | 0.056 | 0.092 |
| Cer(d18:1/25:0) | 0.239 | 0.198 | 0.274 | < 0.001 | < 0.001 |
| Cer(d18:1/26:1) | 0.120 | 0.086 | 0.515 | < 0.001 | < 0.001 |
| Cer(d18:1/26:0) | 0.292 | 0.241 | 0.294 | 0.004 | 0.010 |
| GlcCer(d18:1/16:0) | 0.197 | 0.152 | 0.345 | < 0.001 | < 0.001 |
| GlcCer(d18:1/18:0) | 0.041 | 0.033 | 0.302 | < 0.001 | < 0.001 |
| GlcCer(d18:1/20:0) | 0.034 | 0.028 | 0.266 | < 0.001 | < 0.001 |
| GlcCer(d18:1/22:0) | 0.240 | 0.223 | 0.099 | 0.107 | 0.165 |
| GlcCer(d18:1/23:0) | 0.132 | 0.116 | 0.178 | 0.006 | 0.012 |
| GlcCer(d18:1/24:1) | 0.275 | 0.219 | 0.315 | < 0.001 | < 0.001 |
| GlcCer(d18:1/24:0) | 0.364 | 0.328 | 0.145 | 0.014 | 0.027 |
| GlcCer(d18:1/26:1) | 0.009 | 0.007 | 0.466 | 0.004 | 0.010 |
| GlcCer(d18:1/26:0) | 0.019 | 0.015 | 0.354 | 0.008 | 0.017 |
| **Sphingomyelins** |  |  |  |  |  |
| SM(d31:1) | 0.077 | 0.072 | 0.103 | 0.039 | 0.068 |
| SM(d31:0) | 0.013 | 0.012 | 0.092 | 0.056 | 0.092 |
| SM(d32:1) | 2.414 | 2.156 | 0.161 | < 0.001 | 0.002 |
| SM(d33:1) | 1.306 | 1.124 | 0.212 | < 0.001 | < 0.001 |
| SM(d34:2) | 4.216 | 4.144 | 0.025 | 0.501 | 0.622 |
| SM(d34:1) | 23.010 | 21.147 | 0.122 | < 0.001 | < 0.001 |
| SM(d35:3) | 0.442 | 0.295 | 0.425 | 0.045 | 0.077 |
| SM(d35:1) | 0.812 | 0.721 | 0.162 | < 0.001 | < 0.001 |
| SM(d36:2) | 2.879 | 2.883 | 0.007 | 0.873 | 0.936 |
| SM(d37:2) | 0.174 | 0.165 | 0.072 | 0.182 | 0.271 |
| SM(d37:1) | 0.550 | 0.497 | 0.143 | 0.004 | 0.009 |
| SM(d38:2) | 1.741 | 1.731 | 0.011 | 0.785 | 0.865 |
| SM(d38:1) | 4.327 | 4.199 | 0.046 | 0.225 | 0.322 |
| SM(d39:2) | 0.311 | 0.309 | 0.013 | 0.780 | 0.865 |
| SM(d40:2) | 6.883 | 7.071 | -0.037 | 0.335 | 0.428 |
| SM(d40:1) | 8.966 | 9.188 | -0.034 | 0.418 | 0.529 |
| SM(d41:2) | 2.800 | 2.781 | 0.017 | 0.670 | 0.795 |
| SM(d41:1) | 3.692 | 3.725 | -0.005 | 0.890 | 0.946 |
| SM(d42:3) | 8.437 | 7.944 | 0.100 | 0.023 | 0.044 |
| SM(d42:2) | 16.454 | 15.276 | 0.111 | 0.003 | 0.007 |
| SM(d42:1) | 7.597 | 7.613 | -0.008 | 0.839 | 0.908 |
| SM(d43:3) | 0.241 | 0.206 | 0.222 | 0.005 | 0.011 |
| SM(d43:2) | 0.776 | 0.660 | 0.240 | < 0.001 | < 0.001 |
| SM(d43:1) | 0.553 | 0.528 | 0.066 | 0.104 | 0.163 |
| SM(d44:2) | 0.480 | 0.452 | 0.092 | 0.260 | 0.356 |
| SM(d44:1) | 0.235 | 0.228 | 0.036 | 0.648 | 0.779 |
| SM(d44:0) | 0.055 | 0.052 | 0.071 | 0.233 | 0.330 |
| SM(d45:1) | 0.039 | 0.039 | 0.078 | 0.323 | 0.418 |
| SM(d45:0) | 0.065 | 0.064 | 0.008 | 0.941 | 0.978 |
| SM(d33:3) | 0.682 | 0.473 | 0.427 | 0.025 | 0.047 |
| SM(d36:1) | 5.548 | 5.097 | 0.128 | 0.002 | 0.006 |
| SM(d39:3) | 0.266 | 0.211 | 0.206 | 0.204 | 0.300 |
| SM(d39:1) | 1.536 | 1.522 | 0.019 | 0.675 | 0.795 |
| SM(d40:3) | 0.964 | 0.895 | 0.120 | 0.072 | 0.116 |
| **Keto Acids** |  |  |  |  |  |
| KIV | 12.456 | 12.275 | 0.014 | 0.697 | 0.813 |
| KIC | 29.590 | 32.266 | -0.147 | 0.003 | 0.008 |
| KMV | 20.160 | 20.874 | -0.057 | 0.248 | 0.348 |
| **Other** |  |  |  |  |  |
| 3-HIB | 14.918 | 15.697 | -0.025 | 0.710 | 0.813 |

**Table S4.** Differences in clinical analyte levels between the IPF and control cohorts.

| **Clinical analyte** | **IPF cohort  (mean)** | **Control cohort  (mean)** | **Log_2_-fold change (IPF vs control)** | **Raw *P* value** | **FDR-corrected *P* value** |
| --- | --- | --- | --- | --- | --- |
| Total cholesterol | 200.490 | 170.730 | 0.242 | < 0.001 | < 0.001 |
| Glucose | 108.800 | 122.460 | -0.127 | 0.006 | 0.01 |
| Ketones | 127.918 | 100.667 | 0.269 | 0.045 | 0.07 |
| Lactate | 2.037 | 1.989 | 0.108 | 0.095 | 0.11 |
| Non-esterified fatty acids (NEFA) | 0.518 | 0.321 | 0.751 | < 0.001 | < 0.001 |
| Triglycerides | 154.083 | 174.570 | -0.145 | 0.081 | 0.11 |
| Glycerol | 0.736 | 0.587 | 0.255 | 0.003 | 0.01 |
| 3-Hydroxybutyrate | 86.154 | 69.551 | 0.245 | 0.101 | 0.11 |
| High-density lipoprotein cholesterol | 52.356 | 57.408 | -0.140 | 0.004 | 0.01 |
| Low-density lipoprotein cholesterol | 100.252 | 107.242 | -0.078 | 0.15 | 0.15 |

**Table S5.** Differences in metabolite and clinical analyte levels between patients with IPF who were and were not using anti-fibrotic therapy at enrollment. Metabolites and clinical analytes with absolute fold-change ≥30% (|log_2_FC|>0.38) and corrected p<0.05 in patients with IPF vs controls are shown.

|  | **Using anti-fibrotic treatment (mean)** | **Not using anti-fibrotic treatment (mean)** | **Log_2_-fold change** | **Raw *P* value** | **FDR-corrected *P* value** |
| --- | --- | --- | --- | --- | --- |
| **Acylcarnitines** |  |  |  |  |  |
| C10 | 0.193 | 0.271 | -0.189 | 0.063 | 0.61 |
| C14:1 | 0.070 | 0.086 | -0.169 | 0.057 | 0.61 |
| C14 | 0.032 | 0.036 | -0.103 | 0.082 | 0.61 |
| C16:1 | 0.027 | 0.031 | -0.150 | 0.074 | 0.61 |
| C16 | 0.108 | 0.101 | 0.028 | 0.59 | 0.92 |
| C18:2 | 0.092 | 0.091 | 0.009 | 0.89 | 0.95 |
| C18:1 | 0.155 | 0.153 | -0.015 | 0.80 | 0.92 |
| C20:4 | 0.008 | 0.008 | 0.061 | 0.51 | 0.92 |
| C22 | 0.004 | 0.004 | 0.173 | 0.22 | 0.80 |
| **Amino Acids** |  |  |  |  |  |
| Orn | 96.512 | 94.041 | 0.028 | 0.55 | 0.92 |
| **Ceramides** |  |  |  |  |  |
| Cer(d18:1/18:0) | 0.134 | 0.131 | 0.019 | 0.72 | 0.92 |
| Cer(d18:1/20:0) | 0.147 | 0.148 | -0.001 | 0.99 | 0.99 |
| Cer(d18:1/26:1) | 0.121 | 0.119 | 0.001 | 0.99 | 0.99 |
| GlcCer(d18:1/26:1) | 0.009 | 0.009 | 0.119 | 0.46 | 0.92 |
| **Sphingomyelins** |  |  |  |  |  |
| SM(d33:3) | 0.732 | 0.618 | 0.072 | 0.71 | 0.92 |
| **Clinical Analytes** |  |  |  |  |  |
| NEFA | 0.494 | 0.548 | -0.200 | 0.077 | 0.61 |

**Table S6.** Associations of circulating metabolites with DLco % predicted at enrollment in patients with IPF, unadjusted and adjusted for use of anti-fibrotic therapy at enrollment.

| **Metabolite** | **Unadjusted** | | | | **Adjusted for use of anti-fibrotic treatment** | | | |
| --- | --- | --- | --- | --- | --- | --- | --- | --- |
|  | **Difference in disease severity measure per unit change in log_2_-concentration of metabolite** | **Difference in log_2_-concentration of metabolite per 5-unit change in disease severity measure** | **Raw *P* value** | **FDR-corrected *P* value** | **Difference in disease severity measure per unit change in log_2_-concentration of metabolite** | **Difference in log_2_-concentration of metabolite per 5-unit change in disease severity measure** | **Raw *P* value** | **FDR-corrected *P* value** |
| **Acylcarnitines** |  |  |  |  |  |  |  |  |
| C2 | 0.394 | 0.003 | 0.80 | 0.96 | 0.302 | 0.002 | 0.85 | 0.96 |
| C3 | 2.817 | 0.025 | 0.039 | 0.17 | 2.750 | 0.024 | 0.047 | 0.18 |
| C4/Ci4 | -0.142 | -0.002 | 0.90 | 0.97 | -0.241 | -0.003 | 0.84 | 0.96 |
| C5:1 | 4.804 | 0.014 | 0.05 | 0.19 | 4.845 | 0.014 | 0.047 | 0.18 |
| C5 | 2.974 | 0.033 | 0.02 | 0.093 | 2.933 | 0.033 | 0.017 | 0.10 |
| C4-OH | -0.065 | -0.002 | 0.94 | 0.97 | -0.100 | -0.002 | 0.91 | 0.96 |
| C6 | -1.442 | -0.027 | 0.13 | 0.32 | -1.497 | -0.027 | 0.12 | 0.29 |
| C5-OH/C3-DC | 3.948 | 0.031 | 0.006 | 0.061 | 3.895 | 0.030 | 0.008 | 0.068 |
| C4-DC/Ci4-DC | 2.971 | 0.020 | 0.062 | 0.19 | 2.943 | 0.019 | 0.066 | 0.20 |
| C8:1 | 0.028 | 0.000 | 0.98 | 0.99 | -0.000 | -0.000 | 1.00 | 1.00 |
| C8 | -2.881 | -0.049 | 0.003 | 0.049 | -2.951 | -0.050 | 0.003 | 0.042 |
| C5-DC | 0.559 | 0.005 | 0.68 | 0.88 | 0.503 | 0.005 | 0.71 | 0.90 |
| C8:1-OH/C6:1-DC | 0.236 | 0.002 | 0.86 | 0.97 | 0.227 | 0.002 | 0.87 | 0.96 |
| C6-DC/C8-OH | -1.224 | -0.014 | 0.32 | 0.56 | -1.251 | -0.014 | 0.31 | 0.54 |
| C10:3 | -0.164 | -0.002 | 0.88 | 0.97 | -0.172 | -0.002 | 0.87 | 0.96 |
| C10:2 | -2.365 | -0.027 | 0.049 | 0.19 | -2.398 | -0.028 | 0.047 | 0.18 |
| C10:1 | -4.241 | -0.051 | < 0.001 | 0.033 | -4.313 | -0.052 | < 0.001 | 0.029 |
| C10 | -2.669 | -0.053 | 0.004 | 0.049 | -2.737 | -0.053 | 0.003 | 0.042 |
| C7-DC | -0.771 | -0.030 | 0.24 | 0.47 | -0.751 | -0.029 | 0.25 | 0.50 |
| C8:1-DC | -2.641 | -0.030 | 0.031 | 0.16 | -2.680 | -0.030 | 0.029 | 0.15 |
| C10-OH/C8-DC | -3.961 | -0.038 | 0.003 | 0.049 | -4.006 | -0.038 | 0.003 | 0.042 |
| C12:1 | -1.859 | -0.020 | 0.14 | 0.33 | -1.925 | -0.020 | 0.13 | 0.31 |
| C12 | -2.273 | -0.032 | 0.039 | 0.17 | -2.333 | -0.032 | 0.035 | 0.17 |
| C12-OH/C10-DC | -3.479 | -0.033 | 0.008 | 0.071 | -3.542 | -0.034 | 0.008 | 0.068 |
| C14:2 | -3.093 | -0.047 | 0.003 | 0.049 | -3.164 | -0.048 | 0.003 | 0.042 |
| C14:1 | -2.103 | -0.031 | 0.047 | 0.19 | -2.179 | -0.032 | 0.041 | 0.18 |
| C14 | -1.444 | -0.010 | 0.36 | 0.61 | -1.498 | -0.010 | 0.35 | 0.60 |
| C14:1-OH | -0.968 | -0.007 | 0.52 | 0.78 | -1.013 | -0.008 | 0.50 | 0.75 |
| C14-OH/C12-DC | 0.249 | 0.003 | 0.85 | 0.97 | 0.203 | 0.002 | 0.88 | 0.96 |
| C16:2 | -1.418 | -0.021 | 0.18 | 0.39 | -1.457 | -0.022 | 0.17 | 0.39 |
| C16:1 | -1.513 | -0.020 | 0.18 | 0.39 | -1.571 | -0.021 | 0.17 | 0.38 |
| C16 | 0.721 | 0.004 | 0.70 | 0.89 | 0.763 | 0.004 | 0.68 | 0.89 |
| C16:1-OH/C14:1-DC | -0.783 | -0.009 | 0.53 | 0.78 | -0.799 | -0.009 | 0.52 | 0.77 |
| C16-OH/C14-DC | 0.425 | 0.005 | 0.72 | 0.90 | 0.420 | 0.005 | 0.73 | 0.90 |
| C18:2 | -0.241 | -0.002 | 0.87 | 0.97 | -0.259 | -0.002 | 0.87 | 0.96 |
| C18:1 | -0.165 | -0.001 | 0.92 | 0.97 | -0.179 | -0.001 | 0.91 | 0.96 |
| C18 | 0.236 | 0.001 | 0.90 | 0.97 | 0.222 | 0.001 | 0.90 | 0.96 |
| C18:2-OH | 0.656 | 0.008 | 0.58 | 0.80 | 0.730 | 0.009 | 0.54 | 0.79 |
| C18:1-OH/C16:1-DC | -1.713 | -0.031 | 0.074 | 0.20 | -1.754 | -0.032 | 0.069 | 0.20 |
| C18-OH/C16-DC | -1.442 | -0.014 | 0.27 | 0.49 | -1.455 | -0.015 | 0.26 | 0.50 |
| C20:4 | 1.251 | 0.021 | 0.22 | 0.44 | 1.246 | 0.020 | 0.22 | 0.45 |
| C20 | -0.423 | -0.014 | 0.56 | 0.80 | -0.420 | -0.013 | 0.57 | 0.80 |
| C18:1-DC | -1.868 | -0.027 | 0.085 | 0.23 | -1.843 | -0.026 | 0.09 | 0.240 |
| C20-OH/C18-DC | -0.507 | -0.008 | 0.63 | 0.83 | -0.527 | -0.008 | 0.61 | 0.82 |
| C22 | -0.032 | -0.001 | 0.96 | 0.98 | -0.011 | -0.000 | 0.99 | 1.00 |
| **Amino Acids** |  |  |  |  |  |  |  |  |
| Gly | -0.895 | -0.002 | 0.74 | 0.90 | -0.826 | -0.002 | 0.76 | 0.93 |
| Ala | 4.196 | 0.011 | 0.094 | 0.24 | 4.194 | 0.011 | 0.096 | 0.25 |
| Ser | 3.183 | 0.010 | 0.18 | 0.39 | 3.156 | 0.009 | 0.18 | 0.40 |
| Pro | 4.249 | 0.018 | 0.035 | 0.17 | 4.286 | 0.018 | 0.034 | 0.17 |
| Val | 8.646 | 0.020 | 0.001 | 0.049 | 8.622 | 0.020 | 0.001 | 0.042 |
| Leu/Ile | 6.930 | 0.022 | 0.003 | 0.049 | 6.948 | 0.022 | 0.003 | 0.042 |
| Met | 4.233 | 0.014 | 0.064 | 0.19 | 4.260 | 0.014 | 0.064 | 0.20 |
| His | 8.118 | 0.012 | 0.015 | 0.093 | 8.129 | 0.012 | 0.015 | 0.10 |
| Phe | 3.187 | 0.005 | 0.31 | 0.55 | 3.248 | 0.005 | 0.31 | 0.54 |
| Tyr | 5.841 | 0.018 | 0.012 | 0.093 | 6.053 | 0.018 | 0.010 | 0.079 |
| Asx | -0.216 | -0.001 | 0.92 | 0.97 | -0.206 | -0.001 | 0.93 | 0.97 |
| Glx | 5.335 | 0.019 | 0.013 | 0.093 | 5.599 | 0.019 | 0.012 | 0.089 |
| Orn | 4.467 | 0.019 | 0.023 | 0.13 | 4.547 | 0.020 | 0.022 | 0.12 |
| Cit | -0.541 | -0.004 | 0.73 | 0.90 | -0.577 | -0.004 | 0.72 | 0.90 |
| Arg | -1.323 | -0.008 | 0.43 | 0.69 | -1.373 | -0.008 | 0.42 | 0.68 |
| **Ceramides** |  |  |  |  |  |  |  |  |
| Cer(d18:1/14:0) | -0.305 | -0.003 | 0.83 | 0.97 | -0.272 | -0.002 | 0.85 | 0.96 |
| Cer(d18:1/16:0) | -6.504 | -0.024 | 0.002 | 0.049 | -6.484 | -0.024 | 0.002 | 0.042 |
| Cer(d18:1/18:0) | -4.830 | -0.028 | 0.005 | 0.050 | -4.791 | -0.027 | 0.005 | 0.056 |
| Cer(d18:1/20:1) | -1.265 | -0.013 | 0.33 | 0.57 | -1.276 | -0.013 | 0.33 | 0.56 |
| Cer(d18:1/20:0) | -2.274 | -0.028 | 0.051 | 0.19 | -2.264 | -0.028 | 0.053 | 0.19 |
| Cer(d18:1/22:0) | -4.036 | -0.018 | 0.036 | 0.17 | -3.977 | -0.018 | 0.040 | 0.18 |
| Cer(d18:1/23:0) | -2.350 | -0.011 | 0.22 | 0.44 | -2.345 | -0.011 | 0.23 | 0.45 |
| Cer(d18:1/24:1) | -3.708 | -0.016 | 0.057 | 0.19 | -3.659 | -0.016 | 0.061 | 0.20 |
| Cer(d18:1/24:0) | -1.465 | -0.007 | 0.45 | 0.72 | -1.348 | -0.006 | 0.49 | 0.75 |
| Cer(d18:1/25:0) | -1.220 | -0.007 | 0.46 | 0.73 | -1.188 | -0.007 | 0.48 | 0.75 |
| Cer(d18:1/26:1) | -0.568 | -0.009 | 0.58 | 0.80 | -0.553 | -0.009 | 0.59 | 0.80 |
| Cer(d18:1/26:0) | 0.078 | 0.002 | 0.93 | 0.97 | 0.110 | 0.002 | 0.91 | 0.96 |
| GlcCer(d18:1/16:0) | -4.947 | -0.031 | 0.002 | 0.049 | -4.923 | -0.031 | 0.002 | 0.042 |
| GlcCer(d18:1/18:0) | -2.843 | -0.021 | 0.061 | 0.19 | -2.823 | -0.020 | 0.064 | 0.20 |
| GlcCer(d18:1/20:0) | -3.612 | -0.027 | 0.016 | 0.09 | -3.593 | -0.027 | 0.016 | 0.10 |
| GlcCer(d18:1/22:0) | -2.802 | -0.020 | 0.066 | 0.19 | -2.784 | -0.020 | 0.069 | 0.20 |
| GlcCer(d18:1/23:0) | -2.783 | -0.022 | 0.055 | 0.19 | -2.806 | -0.022 | 0.054 | 0.19 |
| GlcCer(d18:1/24:1) | -3.544 | -0.024 | 0.024 | 0.13 | -3.560 | -0.024 | 0.024 | 0.13 |
| GlcCer(d18:1/24:0) | -3.163 | -0.021 | 0.045 | 0.19 | -3.161 | -0.021 | 0.046 | 0.18 |
| GlcCer(d18:1/26:1) | -0.085 | -0.004 | 0.89 | 0.97 | -0.075 | -0.004 | 0.90 | 0.96 |
| GlcCer(d18:1/26:0) | -0.597 | -0.021 | 0.39 | 0.64 | -0.607 | -0.021 | 0.39 | 0.63 |
| **Sphingomyelins** |  |  |  |  |  |  |  |  |
| SM(d31:1) | -0.012 | -0.000 | 1.00 | 1.00 | 0.024 | 0.000 | 0.99 | 1.00 |
| SM(d31:0) | -0.431 | -0.002 | 0.82 | 0.97 | -0.337 | -0.002 | 0.86 | 0.96 |
| SM(d32:1) | 1.049 | 0.004 | 0.61 | 0.83 | 1.128 | 0.004 | 0.59 | 0.80 |
| SM(d33:1) | -3.039 | -0.011 | 0.16 | 0.38 | -2.975 | -0.011 | 0.17 | 0.39 |
| SM(d34:2) | -4.794 | -0.013 | 0.053 | 0.19 | -4.889 | -0.013 | 0.052 | 0.19 |
| SM(d34:1) | -8.620 | -0.016 | 0.004 | 0.05 | -8.555 | -0.016 | 0.005 | 0.056 |
| SM(d35:3) | -0.153 | -0.014 | 0.72 | 0.90 | -0.148 | -0.013 | 0.73 | 0.90 |
| SM(d35:1) | -3.063 | -0.010 | 0.17 | 0.39 | -2.967 | -0.010 | 0.19 | 0.41 |
| SM(d36:2) | -4.019 | -0.015 | 0.058 | 0.19 | -4.002 | -0.015 | 0.06 | 0.20 |
| SM(d37:2) | -1.976 | -0.011 | 0.25 | 0.48 | -1.917 | -0.011 | 0.27 | 0.504 |
| SM(d37:1) | -0.775 | -0.004 | 0.68 | 0.88 | -0.698 | -0.003 | 0.72 | 0.90 |
| SM(d38:2) | -4.269 | -0.013 | 0.068 | 0.19 | -4.230 | -0.013 | 0.073 | 0.20 |
| SM(d38:1) | -0.119 | -0.000 | 0.96 | 0.98 | 0.018 | 0.000 | 0.99 | 1.00 |
| SM(d39:2) | -1.385 | -0.006 | 0.49 | 0.76 | -1.395 | -0.006 | 0.49 | 0.75 |
| SM(d40:2) | -2.334 | -0.006 | 0.35 | 0.59 | -2.303 | -0.006 | 0.36 | 0.60 |
| SM(d40:1) | -0.184 | -0.001 | 0.94 | 0.97 | -0.101 | -0.000 | 0.96 | 1.00 |
| SM(d41:2) | -1.351 | -0.004 | 0.58 | 0.80 | -1.366 | -0.004 | 0.57 | 0.80 |
| SM(d41:1) | 0.910 | 0.003 | 0.71 | 0.90 | 0.966 | 0.003 | 0.69 | 0.90 |
| SM(d42:3) | -5.324 | -0.018 | 0.016 | 0.093 | -5.355 | -0.018 | 0.02 | 0.10 |
| SM(d42:2) | -3.507 | -0.009 | 0.17 | 0.39 | -3.442 | -0.009 | 0.18 | 0.40 |
| SM(d42:1) | 1.406 | 0.005 | 0.53 | 0.78 | 1.521 | 0.005 | 0.50 | 0.75 |
| SM(d43:3) | -1.482 | -0.018 | 0.21 | 0.44 | -1.449 | -0.017 | 0.22 | 0.45 |
| SM(d43:2) | 1.069 | 0.005 | 0.59 | 0.81 | 1.200 | 0.005 | 0.55 | 0.79 |
| SM(d43:1) | 3.444 | 0.011 | 0.14 | 0.33 | 3.656 | 0.011 | 0.12 | 0.29 |
| SM(d44:2) | 0.742 | 0.009 | 0.52 | 0.78 | 0.747 | 0.009 | 0.52 | 0.77 |
| SM(d44:1) | 1.257 | 0.015 | 0.30 | 0.54 | 1.290 | 0.015 | 0.29 | 0.52 |
| SM(d44:0) | 0.147 | 0.001 | 0.92 | 0.97 | 0.247 | 0.002 | 0.87 | 0.96 |
| SM(d45:1) | -0.584 | -0.007 | 0.63 | 0.83 | -0.562 | -0.006 | 0.64 | 0.85 |
| SM(d45:0) | -0.788 | -0.016 | 0.38 | 0.63 | -0.780 | -0.016 | 0.39 | 0.63 |
| SM(d33:3) | -0.271 | -0.020 | 0.58 | 0.80 | -0.268 | -0.019 | 0.58 | 0.80 |
| SM(d36:1) | -2.654 | -0.009 | 0.25 | 0.47 | -2.543 | -0.008 | 0.27 | 0.504 |
| SM(d39:3) | -0.134 | -0.007 | 0.81 | 0.97 | -0.129 | -0.007 | 0.82 | 0.96 |
| SM(d39:1) | 2.221 | 0.009 | 0.29 | 0.52 | 2.262 | 0.009 | 0.28 | 0.509 |
| SM(d40:3) | -2.612 | -0.021 | 0.068 | 0.19 | -2.598 | -0.021 | 0.071 | 0.20 |
| **Keto Acids** |  |  |  |  |  |  |  |  |
| KIV | 3.098 | 0.008 | 0.22 | 0.44 | 3.001 | 0.008 | 0.24 | 0.48 |
| KIC | 3.389 | 0.017 | 0.062 | 0.19 | 3.337 | 0.017 | 0.067 | 0.20 |
| KMV | 2.916 | 0.014 | 0.12 | 0.30 | 2.879 | 0.014 | 0.13 | 0.305 |
| **Other** |  |  |  |  |  |  |  |  |
| 3-HIB | 3.923 | 0.031 | 0.007 | 0.06 | 3.975 | 0.031 | 0.007 | 0.067 |

**Table S7.** Association of circulating metabolites with FVC % predicted at enrollment in patients with IPF, unadjusted and adjusted for use of anti-fibrotic therapy at enrollment.

| **Metabolite** | **Unadjusted** | | | | | | **Adjusted for use of anti-fibrotic treatment** | | | |
| --- | --- | --- | --- | --- | --- | --- | --- | --- | --- | --- |
|  | **Difference in disease severity measure per unit change in log_2_-concentration of metabolite** | **Difference in log_2_-concentration of metabolite per 5-unit change in disease severity measure** | | **Raw *P* value** | **FDR-corrected *P* value** | | **Difference in disease severity measure per unit change in log_2_-concentration of metabolite** | **Difference in log_2_-concentration of metabolite per 5-unit change in disease severity measure** | **Raw  *P* value** | **FDR-corrected *P* value** |
| **Acylcarnitines** |  |  |  | | |  |  |  |  |  |
| C2 | -0.773 | -0.004 | 0.67 | | | 0.95 | -0.925 | -0.005 | 0.61 | 0.92 |
| C3 | 3.242 | 0.021 | 0.045 | | | 0.49 | 3.189 | 0.020 | 0.051 | 0.51 |
| C4/Ci4 | 2.915 | 0.025 | 0.036 | | | 0.43 | 2.874 | 0.024 | 0.041 | 0.49 |
| C5:1 | 8.949 | 0.018 | 0.002 | | | 0.20 | 9.031 | 0.018 | 0.002 | 0.19 |
| C5 | 3.093 | 0.025 | 0.033 | | | 0.43 | 3.046 | 0.024 | 0.036 | 0.48 |
| C4-OH | -1.110 | -0.019 | 0.27 | | | 0.83 | -1.165 | -0.019 | 0.25 | 0.80 |
| C6 | -0.802 | -0.011 | 0.48 | | | 0.83 | -0.868 | -0.011 | 0.45 | 0.82 |
| C5-OH/C3-DC | 4.124 | 0.023 | 0.016 | | | 0.40 | 4.087 | 0.023 | 0.019 | 0.45 |
| C4-DC/Ci4-DC | 1.313 | 0.006 | 0.49 | | | 0.83 | 1.310 | 0.006 | 0.49 | 0.83 |
| C8:1 | 0.086 | 0.001 | 0.95 | | | 0.99 | 0.051 | 0.000 | 0.97 | 0.99 |
| C8 | -1.089 | -0.013 | 0.35 | | | 0.83 | -1.166 | -0.014 | 0.33 | 0.82 |
| C5-DC | 4.455 | 0.029 | 0.006 | | | 0.33 | 4.413 | 0.028 | 0.006 | 0.38 |
| C8:1-OH/C6:1-DC | 2.790 | 0.018 | 0.081 | | | 0.64 | 2.772 | 0.018 | 0.084 | 0.66 |
| C6-DC/C8-OH | 0.270 | 0.002 | 0.85 | | | 0.98 | 0.251 | 0.002 | 0.86 | 0.98 |
| C10:3 | 0.743 | 0.008 | 0.56 | | | 0.90 | 0.750 | 0.008 | 0.56 | 0.88 |
| C10:2 | 0.255 | 0.002 | 0.86 | | | 0.98 | 0.255 | 0.002 | 0.86 | 0.98 |
| C10:1 | -2.668 | -0.023 | 0.056 | | | 0.55 | -2.755 | -0.024 | 0.050 | 0.508 |
| C10 | -0.916 | -0.013 | 0.40 | | | 0.83 | -0.990 | -0.014 | 0.37 | 0.82 |
| C7-DC | -0.539 | -0.015 | 0.49 | | | 0.83 | -0.531 | -0.015 | 0.50 | 0.83 |
| C8:1-DC | -0.514 | -0.004 | 0.72 | | | 0.98 | -0.550 | -0.004 | 0.706 | 0.95 |
| C10-OH/C8-DC | -1.736 | -0.012 | 0.27 | | | 0.83 | -1.780 | -0.012 | 0.26 | 0.80 |
| C12:1 | -1.911 | -0.014 | 0.20 | | | 0.83 | -2.007 | -0.015 | 0.18 | 0.80 |
| C12 | -1.112 | -0.011 | 0.39 | | | 0.83 | -1.188 | -0.012 | 0.37 | 0.82 |
| C12-OH/C10-DC | -2.340 | -0.016 | 0.14 | | | 0.77 | -2.416 | -0.016 | 0.13 | 0.72 |
| C14:2 | -2.650 | -0.029 | 0.033 | | | 0.43 | -2.741 | -0.029 | 0.028 | 0.48 |
| C14:1 | -2.130 | -0.023 | 0.090 | | | 0.67 | -2.233 | -0.023 | 0.078 | 0.66 |
| C14 | -1.745 | -0.008 | 0.35 | | | 0.83 | -1.866 | -0.009 | 0.33 | 0.82 |
| C14:1-OH | -2.709 | -0.015 | 0.12 | | | 0.76 | -2.753 | -0.015 | 0.12 | 0.72 |
| C14-OH/C12-DC | -2.719 | -0.020 | 0.075 | | | 0.64 | -2.777 | -0.020 | 0.070 | 0.64 |
| C16:2 | -1.503 | -0.016 | 0.23 | | | 0.83 | -1.559 | -0.017 | 0.22 | 0.80 |
| C16:1 | -1.736 | -0.017 | 0.19 | | | 0.83 | -1.830 | -0.017 | 0.17 | 0.80 |
| C16 | -1.011 | -0.004 | 0.64 | | | 0.95 | -0.969 | -0.003 | 0.66 | 0.94 |
| C16:1-OH/C14:1-DC | 0.161 | 0.001 | 0.91 | | | 0.99 | 0.146 | 0.001 | 0.92 | 0.99 |
| C16-OH/C14-DC | -1.474 | -0.012 | 0.30 | | | 0.83 | -1.460 | -0.012 | 0.302 | 0.82 |
| C18:2 | -1.470 | -0.008 | 0.41 | | | 0.83 | -1.469 | -0.008 | 0.42 | 0.82 |
| C18:1 | -2.114 | -0.010 | 0.26 | | | 0.83 | -2.129 | -0.010 | 0.26 | 0.80 |
| C18 | 0.391 | 0.001 | 0.86 | | | 0.98 | 0.428 | 0.002 | 0.85 | 0.98 |
| C18:2-OH | 0.924 | 0.008 | 0.51 | | | 0.86 | 1.036 | 0.009 | 0.47 | 0.82 |
| C18:1-OH/C16:1-DC | -2.504 | -0.033 | 0.027 | | | 0.43 | -2.570 | -0.033 | 0.024 | 0.48 |
| C18-OH/C16-DC | -1.809 | -0.013 | 0.24 | | | 0.83 | -1.802 | -0.013 | 0.24 | 0.80 |
| C20:4 | 0.906 | 0.011 | 0.45 | | | 0.83 | 0.930 | 0.011 | 0.44 | 0.82 |
| C20 | -0.178 | -0.004 | 0.84 | | | 0.98 | -0.147 | -0.003 | 0.86 | 0.98 |
| C18:1-DC | -1.676 | -0.017 | 0.19 | | | 0.83 | -1.658 | -0.017 | 0.20 | 0.80 |
| C20-OH/C18-DC | 0.081 | 0.001 | 0.95 | | | 0.99 | 0.061 | 0.001 | 0.96 | 0.99 |
| C22 | -0.185 | -0.005 | 0.82 | | | 0.98 | -0.155 | -0.004 | 0.85 | 0.98 |
| **Amino Acids** |  |  |  | | |  |  |  |  |  |
| Gly | -2.809 | -0.005 | 0.38 | | | 0.83 | -2.693 | -0.004 | 0.41 | 0.82 |
| Ala | 0.504 | 0.001 | 0.87 | | | 0.98 | 0.531 | 0.001 | 0.86 | 0.98 |
| Ser | -2.724 | -0.006 | 0.33 | | | 0.83 | -2.768 | -0.006 | 0.33 | 0.82 |
| Pro | 3.922 | 0.012 | 0.10 | | | 0.70 | 3.924 | 0.012 | 0.10 | 0.71 |
| Val | 4.990 | 0.008 | 0.11 | | | 0.75 | 4.989 | 0.008 | 0.12 | 0.72 |
| Leu/Ile | 4.167 | 0.009 | 0.13 | | | 0.76 | 4.191 | 0.009 | 0.13 | 0.72 |
| Met | -0.796 | -0.002 | 0.77 | | | 0.98 | -0.889 | -0.002 | 0.75 | 0.97 |
| His | 4.863 | 0.005 | 0.22 | | | 0.83 | 4.836 | 0.005 | 0.22 | 0.80 |
| Phe | 3.096 | 0.004 | 0.40 | | | 0.83 | 2.987 | 0.004 | 0.43 | 0.82 |
| Tyr | 3.614 | 0.008 | 0.19 | | | 0.83 | 3.686 | 0.008 | 0.19 | 0.80 |
| Asx | -3.102 | -0.007 | 0.25 | | | 0.83 | -3.097 | -0.007 | 0.25 | 0.80 |
| Glx | 1.438 | 0.004 | 0.57 | | | 0.90 | 1.629 | 0.004 | 0.54 | 0.87 |
| Orn | 5.569 | 0.017 | 0.017 | | | 0.40 | 5.646 | 0.017 | 0.016 | 0.45 |
| Cit | 4.622 | 0.022 | 0.014 | | | 0.40 | 4.602 | 0.021 | 0.015 | 0.45 |
| Arg | 0.285 | 0.001 | 0.89 | | | 0.99 | 0.187 | 0.001 | 0.93 | 0.99 |
| **Ceramides** |  |  |  | | |  |  |  |  |  |
| Cer(d18:1/14:0) | 2.287 | 0.014 | 0.17 | | | 0.83 | 2.315 | 0.014 | 0.17 | 0.80 |
| Cer(d18:1/16:0) | -1.954 | -0.005 | 0.44 | | | 0.83 | -1.917 | -0.005 | 0.45 | 0.82 |
| Cer(d18:1/18:0) | -1.781 | -0.007 | 0.38 | | | 0.83 | -1.754 | -0.007 | 0.39 | 0.82 |
| Cer(d18:1/20:1) | -0.962 | -0.007 | 0.53 | | | 0.86 | -0.968 | -0.007 | 0.53 | 0.86 |
| Cer(d18:1/20:0) | -0.551 | -0.005 | 0.69 | | | 0.97 | -0.549 | -0.005 | 0.69 | 0.95 |
| Cer(d18:1/22:0) | -1.919 | -0.006 | 0.40 | | | 0.83 | -1.834 | -0.006 | 0.42 | 0.82 |
| Cer(d18:1/23:0) | 0.733 | 0.002 | 0.75 | | | 0.98 | 0.792 | 0.003 | 0.73 | 0.97 |
| Cer(d18:1/24:1) | -0.598 | -0.002 | 0.80 | | | 0.98 | -0.528 | -0.002 | 0.82 | 0.98 |
| Cer(d18:1/24:0) | -0.073 | -0.000 | 0.98 | | | 0.99 | 0.066 | 0.000 | 0.98 | 0.99 |
| Cer(d18:1/25:0) | 0.908 | 0.004 | 0.65 | | | 0.95 | 0.942 | 0.004 | 0.63 | 0.94 |
| Cer(d18:1/26:1) | 1.230 | 0.014 | 0.31 | | | 0.83 | 1.237 | 0.014 | 0.31 | 0.82 |
| Cer(d18:1/26:0) | 0.987 | 0.014 | 0.37 | | | 0.83 | 1.009 | 0.014 | 0.36 | 0.82 |
| GlcCer(d18:1/16:0) | -1.836 | -0.008 | 0.34 | | | 0.83 | -1.796 | -0.008 | 0.35 | 0.82 |
| GlcCer(d18:1/18:0) | -0.784 | -0.004 | 0.66 | | | 0.95 | -0.788 | -0.004 | 0.66 | 0.94 |
| GlcCer(d18:1/20:0) | -0.066 | -0.000 | 0.97 | | | 0.99 | -0.055 | -0.000 | 0.98 | 0.99 |
| GlcCer(d18:1/22:0) | -0.248 | -0.001 | 0.89 | | | 0.99 | -0.226 | -0.001 | 0.90 | 0.99 |
| GlcCer(d18:1/23:0) | 0.434 | 0.002 | 0.80 | | | 0.98 | 0.429 | 0.002 | 0.80 | 0.98 |
| GlcCer(d18:1/24:1) | 0.038 | 0.000 | 0.98 | | | 0.99 | 0.050 | 0.000 | 0.98 | 0.99 |
| GlcCer(d18:1/24:0) | 0.564 | 0.003 | 0.76 | | | 0.98 | 0.582 | 0.003 | 0.76 | 0.98 |
| GlcCer(d18:1/26:1) | 0.909 | 0.032 | 0.19 | | | 0.83 | 0.927 | 0.032 | 0.18 | 0.80 |
| GlcCer(d18:1/26:0) | 1.035 | 0.025 | 0.21 | | | 0.83 | 1.020 | 0.025 | 0.22 | 0.80 |
| **Sphingomyelins** |  |  |  | | |  |  |  |  |  |
| SM(d31:1) | 1.713 | 0.006 | 0.44 | | | 0.83 | 1.755 | 0.006 | 0.43 | 0.82 |
| SM(d31:0) | 0.811 | 0.003 | 0.72 | | | 0.98 | 0.902 | 0.003 | 0.69 | 0.95 |
| SM(d32:1) | 5.087 | 0.014 | 0.036 | | | 0.43 | 5.139 | 0.014 | 0.036 | 0.48 |
| SM(d33:1) | 1.443 | 0.004 | 0.58 | | | 0.90 | 1.517 | 0.004 | 0.56 | 0.88 |
| SM(d34:2) | -2.203 | -0.004 | 0.45 | | | 0.83 | -2.367 | -0.004 | 0.43 | 0.82 |
| SM(d34:1) | 0.245 | 0.000 | 0.95 | | | 0.99 | 0.348 | 0.000 | 0.92 | 0.99 |
| SM(d35:3) | -0.088 | -0.006 | 0.86 | | | 0.98 | -0.081 | -0.005 | 0.88 | 0.98 |
| SM(d35:1) | -0.031 | -0.000 | 0.99 | | | 0.99 | 0.090 | 0.000 | 0.97 | 0.99 |
| SM(d36:2) | -2.744 | -0.007 | 0.28 | | | 0.83 | -2.791 | -0.007 | 0.27 | 0.81 |
| SM(d37:2) | -1.666 | -0.007 | 0.42 | | | 0.83 | -1.638 | -0.007 | 0.43 | 0.82 |
| SM(d37:1) | 0.278 | 0.001 | 0.902 | | | 0.99 | 0.353 | 0.001 | 0.88 | 0.98 |
| SM(d38:2) | -3.445 | -0.007 | 0.22 | | | 0.83 | -3.469 | -0.007 | 0.22 | 0.80 |
| SM(d38:1) | 2.296 | 0.004 | 0.43 | | | 0.83 | 2.427 | 0.005 | 0.41 | 0.82 |
| SM(d39:2) | -0.903 | -0.003 | 0.705 | | | 0.98 | -0.948 | -0.003 | 0.69 | 0.95 |
| SM(d40:2) | -0.496 | -0.001 | 0.87 | | | 0.98 | -0.534 | -0.001 | 0.86 | 0.98 |
| SM(d40:1) | 0.563 | 0.001 | 0.83 | | | 0.98 | 0.628 | 0.001 | 0.81 | 0.98 |
| SM(d41:2) | 2.009 | 0.004 | 0.48 | | | 0.83 | 1.973 | 0.004 | 0.49 | 0.83 |
| SM(d41:1) | 2.897 | 0.006 | 0.32 | | | 0.83 | 2.980 | 0.006 | 0.30 | 0.82 |
| SM(d42:3) | -1.145 | -0.003 | 0.66 | | | 0.95 | -1.210 | -0.003 | 0.65 | 0.94 |
| SM(d42:2) | 1.677 | 0.003 | 0.58 | | | 0.90 | 1.729 | 0.003 | 0.57 | 0.88 |
| SM(d42:1) | 2.076 | 0.005 | 0.43 | | | 0.83 | 2.191 | 0.005 | 0.409 | 0.82 |
| SM(d43:3) | 0.095 | 0.001 | 0.95 | | | 0.99 | 0.115 | 0.001 | 0.94 | 0.99 |
| SM(d43:2) | 1.999 | 0.006 | 0.40 | | | 0.83 | 2.092 | 0.006 | 0.38 | 0.82 |
| SM(d43:1) | 2.939 | 0.007 | 0.28 | | | 0.83 | 3.097 | 0.007 | 0.26 | 0.80 |
| SM(d44:2) | 1.609 | 0.014 | 0.24 | | | 0.83 | 1.591 | 0.014 | 0.25 | 0.80 |
| SM(d44:1) | 1.240 | 0.010 | 0.38 | | | 0.83 | 1.247 | 0.010 | 0.39 | 0.82 |
| SM(d44:0) | -0.472 | -0.002 | 0.80 | | | 0.98 | -0.401 | -0.002 | 0.83 | 0.98 |
| SM(d45:1) | 1.073 | 0.009 | 0.45 | | | 0.83 | 1.077 | 0.009 | 0.45 | 0.82 |
| SM(d45:0) | 0.782 | 0.012 | 0.46 | | | 0.83 | 0.788 | 0.012 | 0.46 | 0.82 |
| SM(d33:3) | -0.176 | -0.009 | 0.76 | | | 0.98 | -0.170 | -0.009 | 0.77 | 0.98 |
| SM(d36:1) | -0.187 | -0.000 | 0.95 | | | 0.99 | -0.090 | -0.000 | 0.97 | 0.99 |
| SM(d39:3) | -0.317 | -0.012 | 0.63 | | | 0.95 | -0.311 | -0.012 | 0.64 | 0.94 |
| SM(d39:1) | 3.559 | 0.010 | 0.15 | | | 0.80 | 3.588 | 0.010 | 0.15 | 0.79 |
| SM(d40:3) | -1.070 | -0.006 | 0.53 | | | 0.86 | -1.078 | -0.006 | 0.53 | 0.86 |
| **Keto Acids** |  |  |  | | |  |  |  |  |  |
| KIV | -1.544 | -0.003 | 0.608 | | | 0.93 | -1.703 | -0.003 | 0.57 | 0.88 |
| KIC | 0.024 | 0.000 | 0.99 | | | 0.99 | -0.040 | -0.000 | 0.99 | 0.99 |
| KMV | -0.678 | -0.002 | 0.76 | | | 0.98 | -0.739 | -0.003 | 0.74 | 0.97 |
| **Other** |  |  |  | | |  |  |  |  |  |
| 3-HIB | 2.989 | 0.017 | 0.081 | | | 0.64 | 2.956 | 0.016 | 0.089 | 0.67 |

**Table S8.** Association of circulating metabolites with composite physiologic index at enrollment in patients with IPF unadjusted and adjusted for use of anti-fibrotic therapy at enrollment.

| **Metabolite** | **Unadjusted** | | | | **Adjusted for use of anti-fibrotic treatment** | | | |
| --- | --- | --- | --- | --- | --- | --- | --- | --- |
|  | **Difference in disease severity measure per unit change in log_2_-concentration of metabolite** | **Difference in log_2_-concentration of metabolite per 5-unit change in disease severity measure** | **Raw *P* value** | **FDR-corrected *P* value** | **Difference in disease severity measure per unit change in log_2_-concentration of metabolite** | **Difference in log_2_-concentration of metabolite per 5-unit change in disease severity measure** | **Raw  *P* value** | **FDR-corrected *P* value** |
| **Acylcarnitines** |  |  |  |  |  |  |  |  |
| C2 | -0.284 | -0.003 | 0.814 | 0.923 | -0.185 | -0.002 | 0.880 | 0.944 |
| C3 | -2.124 | -0.031 | 0.049 | 0.233 | -2.085 | -0.030 | 0.056 | 0.247 |
| C4/Ci4 | -0.556 | -0.011 | 0.550 | 0.796 | -0.503 | -0.010 | 0.592 | 0.820 |
| C5:1 | -4.787 | -0.022 | 0.012 | 0.096 | -4.837 | -0.022 | 0.012 | 0.096 |
| C5 | -2.509 | -0.045 | 0.009 | 0.096 | -2.478 | -0.044 | 0.010 | 0.096 |
| C4-OH | 0.203 | 0.008 | 0.762 | 0.915 | 0.241 | 0.009 | 0.721 | 0.885 |
| C6 | 0.912 | 0.027 | 0.225 | 0.487 | 0.963 | 0.028 | 0.203 | 0.448 |
| C5-OH/C3-DC | -3.019 | -0.039 | 0.008 | 0.096 | -2.998 | -0.038 | 0.010 | 0.096 |
| C4-DC/Ci4-DC | -1.892 | -0.020 | 0.132 | 0.366 | -1.898 | -0.020 | 0.133 | 0.360 |
| C8:1 | -0.038 | -0.001 | 0.967 | 0.987 | -0.010 | -0.000 | 0.991 | 0.998 |
| C8 | 1.993 | 0.055 | 0.010 | 0.096 | 2.065 | 0.056 | 0.008 | 0.096 |
| C5-DC | -1.347 | -0.020 | 0.211 | 0.468 | -1.308 | -0.019 | 0.227 | 0.491 |
| C8:1-OH/C6:1-DC | -0.763 | -0.011 | 0.474 | 0.752 | -0.747 | -0.011 | 0.485 | 0.774 |
| C6-DC/C8-OH | 0.955 | 0.017 | 0.323 | 0.588 | 0.972 | 0.018 | 0.316 | 0.579 |
| C10:3 | -0.009 | -0.000 | 0.991 | 0.998 | -0.015 | -0.000 | 0.986 | 0.998 |
| C10:2 | 1.667 | 0.031 | 0.079 | 0.299 | 1.674 | 0.031 | 0.079 | 0.288 |
| C10:1 | 3.080 | 0.060 | < 0.001 | 0.067 | 3.155 | 0.061 | < 0.001 | 0.069 |
| C10 | 1.844 | 0.058 | 0.011 | 0.096 | 1.916 | 0.060 | 0.009 | 0.096 |
| C7-DC | 0.518 | 0.033 | 0.315 | 0.587 | 0.514 | 0.032 | 0.322 | 0.580 |
| C8:1-DC | 1.667 | 0.030 | 0.084 | 0.304 | 1.697 | 0.031 | 0.080 | 0.288 |
| C10-OH/C8-DC | 2.765 | 0.042 | 0.008 | 0.096 | 2.803 | 0.043 | 0.007 | 0.096 |
| C12:1 | 1.237 | 0.021 | 0.212 | 0.468 | 1.306 | 0.022 | 0.191 | 0.448 |
| C12 | 1.580 | 0.035 | 0.068 | 0.271 | 1.644 | 0.036 | 0.060 | 0.254 |
| C12-OH/C10-DC | 2.766 | 0.043 | 0.008 | 0.096 | 2.828 | 0.043 | 0.007 | 0.096 |
| C14:2 | 2.362 | 0.058 | 0.004 | 0.090 | 2.434 | 0.059 | 0.003 | 0.096 |
| C14:1 | 1.554 | 0.037 | 0.063 | 0.260 | 1.629 | 0.039 | 0.053 | 0.247 |
| C14 | 0.960 | 0.010 | 0.443 | 0.725 | 1.046 | 0.011 | 0.408 | 0.675 |
| C14:1-OH | 1.010 | 0.012 | 0.389 | 0.671 | 1.045 | 0.013 | 0.375 | 0.647 |
| C14-OH/C12-DC | 0.218 | 0.004 | 0.831 | 0.926 | 0.252 | 0.004 | 0.805 | 0.921 |
| C16:2 | 1.103 | 0.027 | 0.187 | 0.454 | 1.143 | 0.027 | 0.174 | 0.424 |
| C16:1 | 1.141 | 0.025 | 0.196 | 0.456 | 1.210 | 0.026 | 0.175 | 0.424 |
| C16 | -0.263 | -0.002 | 0.857 | 0.926 | -0.291 | -0.002 | 0.843 | 0.929 |
| C16:1-OH/C14:1-DC | 0.644 | 0.011 | 0.512 | 0.766 | 0.656 | 0.011 | 0.505 | 0.779 |
| C16-OH/C14-DC | 0.268 | 0.005 | 0.775 | 0.915 | 0.254 | 0.005 | 0.788 | 0.921 |
| C18:2 | 0.466 | 0.005 | 0.697 | 0.877 | 0.466 | 0.005 | 0.699 | 0.871 |
| C18:1 | 0.509 | 0.005 | 0.685 | 0.877 | 0.522 | 0.006 | 0.678 | 0.867 |
| C18 | 0.355 | 0.003 | 0.807 | 0.923 | 0.330 | 0.003 | 0.821 | 0.923 |
| C18:2-OH | -0.332 | -0.006 | 0.723 | 0.897 | -0.408 | -0.008 | 0.667 | 0.864 |
| C18:1-OH/C16:1-DC | 1.531 | 0.045 | 0.042 | 0.219 | 1.578 | 0.046 | 0.038 | 0.203 |
| C18-OH/C16-DC | 1.190 | 0.019 | 0.244 | 0.497 | 1.188 | 0.019 | 0.246 | 0.506 |
| C20:4 | -0.839 | -0.022 | 0.292 | 0.552 | -0.862 | -0.023 | 0.283 | 0.561 |
| C20 | 0.370 | 0.019 | 0.517 | 0.766 | 0.350 | 0.018 | 0.543 | 0.793 |
| C18:1-DC | 1.683 | 0.039 | 0.049 | 0.233 | 1.672 | 0.038 | 0.051 | 0.247 |
| C20-OH/C18-DC | 0.461 | 0.012 | 0.575 | 0.796 | 0.477 | 0.012 | 0.563 | 0.797 |
| C22 | 0.226 | 0.014 | 0.670 | 0.876 | 0.205 | 0.012 | 0.700 | 0.871 |
| **Amino Acids** |  |  |  |  |  |  |  |  |
| Gly | 1.211 | 0.004 | 0.573 | 0.796 | 1.116 | 0.004 | 0.606 | 0.820 |
| Ala | -2.335 | -0.010 | 0.238 | 0.497 | -2.374 | -0.010 | 0.232 | 0.494 |
| Ser | -1.198 | -0.006 | 0.521 | 0.766 | -1.168 | -0.006 | 0.533 | 0.793 |
| Pro | -3.617 | -0.024 | 0.023 | 0.141 | -3.623 | -0.024 | 0.023 | 0.136 |
| Val | -6.799 | -0.026 | 0.001 | 0.067 | -6.799 | -0.026 | 0.001 | 0.069 |
| Leu/Ile | -5.556 | -0.028 | 0.002 | 0.089 | -5.567 | -0.028 | 0.002 | 0.090 |
| Met | -2.689 | -0.014 | 0.136 | 0.368 | -2.647 | -0.014 | 0.145 | 0.376 |
| His | -6.478 | -0.016 | 0.014 | 0.096 | -6.456 | -0.016 | 0.014 | 0.099 |
| Phe | -2.690 | -0.007 | 0.276 | 0.542 | -2.611 | -0.007 | 0.299 | 0.564 |
| Tyr | -4.529 | -0.023 | 0.013 | 0.096 | -4.605 | -0.023 | 0.013 | 0.096 |
| Asx | 0.687 | 0.004 | 0.700 | 0.877 | 0.683 | 0.004 | 0.702 | 0.871 |
| Glx | -3.758 | -0.022 | 0.027 | 0.159 | -4.094 | -0.022 | 0.020 | 0.125 |
| Orn | -3.829 | -0.027 | 0.014 | 0.096 | -3.876 | -0.027 | 0.013 | 0.096 |
| Cit | -0.047 | -0.001 | 0.970 | 0.987 | 0.019 | 0.000 | 0.988 | 0.998 |
| Arg | 0.754 | 0.007 | 0.569 | 0.796 | 0.849 | 0.008 | 0.527 | 0.793 |
| **Ceramides** |  |  |  |  |  |  |  |  |
| Cer(d18:1/14:0) | -0.486 | -0.007 | 0.661 | 0.874 | -0.504 | -0.007 | 0.651 | 0.860 |
| Cer(d18:1/16:0) | 4.653 | 0.028 | 0.005 | 0.090 | 4.629 | 0.028 | 0.006 | 0.096 |
| Cer(d18:1/18:0) | 3.287 | 0.030 | 0.015 | 0.097 | 3.279 | 0.030 | 0.015 | 0.102 |
| Cer(d18:1/20:1) | 0.866 | 0.014 | 0.396 | 0.672 | 0.869 | 0.014 | 0.395 | 0.662 |
| Cer(d18:1/20:0) | 1.426 | 0.028 | 0.122 | 0.348 | 1.425 | 0.028 | 0.123 | 0.360 |
| Cer(d18:1/22:0) | 3.100 | 0.023 | 0.041 | 0.219 | 3.049 | 0.022 | 0.046 | 0.236 |
| Cer(d18:1/23:0) | 1.453 | 0.011 | 0.338 | 0.592 | 1.417 | 0.010 | 0.352 | 0.617 |
| Cer(d18:1/24:1) | 2.505 | 0.018 | 0.103 | 0.322 | 2.464 | 0.018 | 0.110 | 0.341 |
| Cer(d18:1/24:0) | 0.995 | 0.007 | 0.516 | 0.766 | 0.911 | 0.006 | 0.555 | 0.796 |
| Cer(d18:1/25:0) | 0.392 | 0.004 | 0.765 | 0.915 | 0.368 | 0.004 | 0.780 | 0.921 |
| Cer(d18:1/26:1) | -0.078 | -0.002 | 0.923 | 0.964 | -0.080 | -0.002 | 0.921 | 0.953 |
| Cer(d18:1/26:0) | -0.381 | -0.012 | 0.601 | 0.822 | -0.395 | -0.012 | 0.590 | 0.820 |
| GlcCer(d18:1/16:0) | 3.576 | 0.036 | 0.005 | 0.090 | 3.551 | 0.036 | 0.006 | 0.096 |
| GlcCer(d18:1/18:0) | 1.955 | 0.023 | 0.103 | 0.322 | 1.966 | 0.023 | 0.102 | 0.329 |
| GlcCer(d18:1/20:0) | 2.267 | 0.027 | 0.055 | 0.250 | 2.260 | 0.027 | 0.056 | 0.247 |
| GlcCer(d18:1/22:0) | 1.937 | 0.022 | 0.108 | 0.329 | 1.921 | 0.022 | 0.112 | 0.341 |
| GlcCer(d18:1/23:0) | 1.682 | 0.022 | 0.142 | 0.369 | 1.685 | 0.022 | 0.143 | 0.376 |
| GlcCer(d18:1/24:1) | 2.310 | 0.025 | 0.063 | 0.260 | 2.303 | 0.025 | 0.065 | 0.258 |
| GlcCer(d18:1/24:0) | 1.925 | 0.021 | 0.123 | 0.348 | 1.914 | 0.021 | 0.126 | 0.360 |
| GlcCer(d18:1/26:1) | -0.187 | -0.015 | 0.685 | 0.877 | -0.199 | -0.016 | 0.668 | 0.864 |
| GlcCer(d18:1/26:0) | 0.097 | 0.005 | 0.861 | 0.926 | 0.109 | 0.006 | 0.844 | 0.929 |
| **Sphingomyelins** |  |  |  |  |  |  |  |  |
| SM(d31:1) | -0.487 | -0.004 | 0.743 | 0.912 | -0.517 | -0.004 | 0.729 | 0.885 |
| SM(d31:0) | 0.249 | 0.002 | 0.869 | 0.926 | 0.189 | 0.001 | 0.901 | 0.953 |
| SM(d32:1) | -1.888 | -0.012 | 0.244 | 0.497 | -1.904 | -0.012 | 0.244 | 0.506 |
| SM(d33:1) | 1.842 | 0.011 | 0.283 | 0.544 | 1.794 | 0.010 | 0.298 | 0.564 |
| SM(d34:2) | 2.872 | 0.013 | 0.142 | 0.369 | 3.034 | 0.013 | 0.127 | 0.360 |
| SM(d34:1) | 5.190 | 0.015 | 0.030 | 0.171 | 5.128 | 0.015 | 0.033 | 0.186 |
| SM(d35:3) | 0.094 | 0.014 | 0.784 | 0.915 | 0.088 | 0.013 | 0.797 | 0.921 |
| SM(d35:1) | 2.061 | 0.011 | 0.246 | 0.497 | 1.988 | 0.011 | 0.266 | 0.537 |
| SM(d36:2) | 2.738 | 0.016 | 0.102 | 0.322 | 2.791 | 0.016 | 0.099 | 0.329 |
| SM(d37:2) | 1.334 | 0.012 | 0.327 | 0.588 | 1.316 | 0.012 | 0.336 | 0.597 |
| SM(d37:1) | 0.307 | 0.002 | 0.838 | 0.926 | 0.254 | 0.002 | 0.866 | 0.937 |
| SM(d38:2) | 3.084 | 0.015 | 0.095 | 0.322 | 3.111 | 0.015 | 0.095 | 0.322 |
| SM(d38:1) | -0.544 | -0.002 | 0.781 | 0.915 | -0.626 | -0.003 | 0.751 | 0.902 |
| SM(d39:2) | 0.805 | 0.005 | 0.614 | 0.830 | 0.834 | 0.006 | 0.602 | 0.820 |
| SM(d40:2) | 1.150 | 0.005 | 0.557 | 0.796 | 1.193 | 0.005 | 0.546 | 0.793 |
| SM(d40:1) | -0.355 | -0.002 | 0.841 | 0.926 | -0.398 | -0.002 | 0.822 | 0.923 |
| SM(d41:2) | 0.307 | 0.001 | 0.871 | 0.926 | 0.334 | 0.002 | 0.861 | 0.937 |
| SM(d41:1) | -1.472 | -0.007 | 0.445 | 0.725 | -1.531 | -0.007 | 0.428 | 0.698 |
| SM(d42:3) | 3.044 | 0.017 | 0.080 | 0.299 | 3.115 | 0.017 | 0.076 | 0.288 |
| SM(d42:2) | 1.371 | 0.006 | 0.501 | 0.766 | 1.346 | 0.005 | 0.510 | 0.779 |
| SM(d42:1) | -1.712 | -0.009 | 0.331 | 0.588 | -1.791 | -0.010 | 0.312 | 0.579 |
| SM(d43:3) | 0.635 | 0.012 | 0.498 | 0.766 | 0.621 | 0.012 | 0.509 | 0.779 |
| SM(d43:2) | -1.317 | -0.009 | 0.401 | 0.672 | -1.381 | -0.009 | 0.383 | 0.650 |
| SM(d43:1) | -2.978 | -0.015 | 0.101 | 0.322 | -3.096 | -0.016 | 0.091 | 0.320 |
| SM(d44:2) | -0.989 | -0.020 | 0.278 | 0.542 | -0.972 | -0.020 | 0.290 | 0.564 |
| SM(d44:1) | -1.219 | -0.023 | 0.199 | 0.456 | -1.222 | -0.023 | 0.201 | 0.448 |
| SM(d44:0) | -0.090 | -0.001 | 0.941 | 0.974 | -0.141 | -0.002 | 0.909 | 0.953 |
| SM(d45:1) | -0.002 | -0.000 | 0.998 | 0.998 | 0.003 | 0.000 | 0.998 | 0.998 |
| SM(d45:0) | 0.076 | 0.003 | 0.915 | 0.963 | 0.072 | 0.002 | 0.919 | 0.953 |
| SM(d33:3) | 0.183 | 0.021 | 0.631 | 0.844 | 0.178 | 0.021 | 0.641 | 0.857 |
| SM(d36:1) | 1.315 | 0.007 | 0.467 | 0.751 | 1.265 | 0.006 | 0.488 | 0.774 |
| SM(d39:3) | 0.115 | 0.010 | 0.795 | 0.918 | 0.110 | 0.010 | 0.804 | 0.921 |
| SM(d39:1) | -2.122 | -0.013 | 0.195 | 0.456 | -2.144 | -0.013 | 0.192 | 0.448 |
| SM(d40:3) | 1.571 | 0.021 | 0.164 | 0.416 | 1.577 | 0.021 | 0.164 | 0.416 |
| **Keto Acids** |  |  |  |  |  |  |  |  |
| KIV | -2.683 | -0.011 | 0.180 | 0.447 | -2.595 | -0.011 | 0.198 | 0.448 |
| KIC | -2.695 | -0.022 | 0.059 | 0.260 | -2.652 | -0.022 | 0.065 | 0.258 |
| KMV | -2.290 | -0.018 | 0.121 | 0.348 | -2.248 | -0.017 | 0.130 | 0.360 |
| **Other** |  |  |  |  |  |  |  |  |
| 3-HIB | -3.239 | -0.042 | 0.004 | 0.090 | -3.234 | -0.040 | 0.005 | 0.096 |

**Fig. S1.** Adjusted univariate associations between metabolites and risk of outcomes. Adjustment variables were age, sex, FVC % predicted, DLco % predicted, and supplemental oxygen use, all assessed at enrollment. Metabolites meeting statistical significance (FDR-corrected P < .05) or clinical significance (hazard ratio <0.67 or >1.5) in unadjusted or adjusted analyses are shown. *Metabolite failed proportional hazards assumptions so hazard ratios at 6, 12, and 24 months are shown. **There was a non-linear relationship between metabolite and outcome so a piecewise linear spline was used; a hazard ratio is shown for each segment.


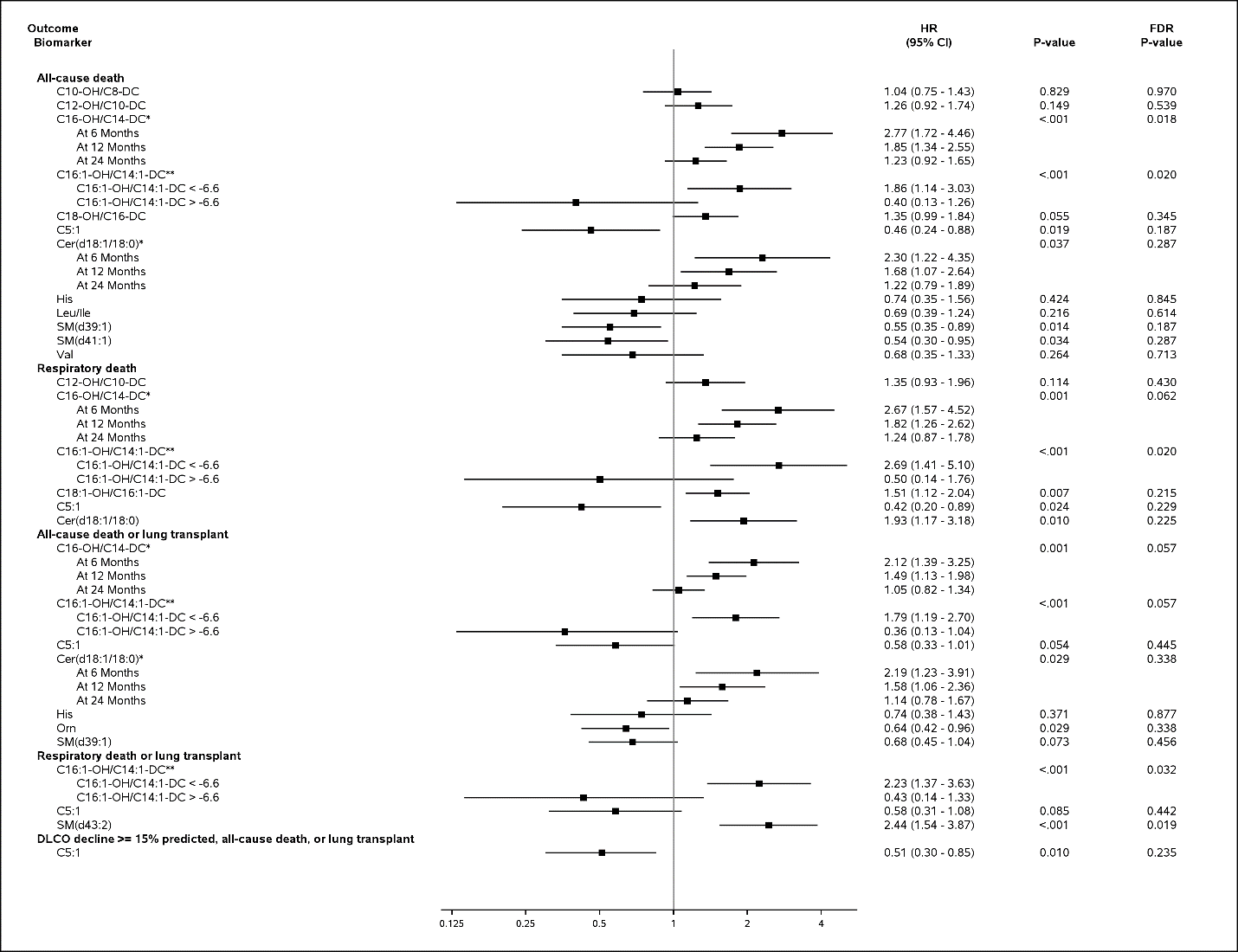

Supplement: Supplementary file 1 — Additional file 1: Table S1. Concentrations of metabolites in the IPF and control cohorts; Table S2. Concentrations of clinical analytes in the IPF and control cohorts; Table S3. Differences in metabolite levels between IPF cases and controls; Table S4. Differences in clinical analyte levels between the IPF and control cohorts; Table S5. Differences in metabolite and clinical analyte levels between patients with IPF who were and were not using anti-fibrotic therapy at enrollment; Table S6. Associations of circulating metabolites with DLco % predicted at enrollment in patients with IPF, unadjusted and adjusted for use of anti-fibrotic therapy at enrollment; Table S7. Association of circulating metabolites with FVC % predicted at enrollment in patients with IPF, unadjusted and adjusted for use of anti-fibrotic therapy at enrollment; Table S8. Association of circulating metabolites with composite physiologic index at enrollment in patients with IPF unadjusted and adjusted for use of anti-fibrotic therapy at enrollment; Figure S1. Adjusted univariate associations between metabolites and risk of outcomes [file 12931_2023_2644_MOESM1_ESM.docx]
